# Standardized generation of human iPSC-derived hematopoietic organoids and macrophages utilizing a benchtop bioreactor platform under fully defined conditions

**DOI:** 10.1186/s13287-024-03785-2

**Published:** 2024-06-18

**Authors:** Mania Ackermann, Fawaz Saleh, Shifaa M. Abdin, Anna Rafiei Hashtchin, Ingrid Gensch, Julia Golgath, Marco Carvalho Oliveira, Ariane H. H. Nguyen, Svenja Gaedcke, Arno Fenske, Mi-Sun Jang, Adan C. Jirmo, Markus Abeln, Gesine Hansen, Nico Lachmann

**Affiliations:** 1 Fraunhofer Institute for Toxicology and Experimental Medicine (ITEM), Hannover, Germany; 2 Department for Pediatric Pneumology, Allergology and Neonatology, Hannover Medical School, Carl-Neuberg-Str. 1, Hannover, Germany; 3 Present Address: Stem Cell Modelling of Development and Disease Group, Max Delbrück Center for Molecular Medicine, Berlin, Germany; 4 Biomedical Research in Endstage and Obstructive Lung Disease Hannover (BREATH), German Center for Lung Research (DZL), Hannover Medical School, Hannover, Germany; 5 Institute of Clinical Biochemistry, Hannover Medical School, Hannover, Germany; 6 RESIST, Cluster of Excellence, Hannover Medical School, Hannover, Germany; 7 Regenerative Biology to Reconstructive Therapy (REBIRTH) Center for Translational and Regenerative Medicine, Hannover Medical School, Hannover, Germany; 8 Department of Respiratory Medicine and Infectious Disease, Hannover Medical School, Hannover, Germany

**Keywords:** Organoids, Hematopoiesis, hiPSC, Macrophages, Up-scaling, Bioreactor, Drug screening, Cell manufacturing

## Abstract

**Background:**

There is a significant demand for intermediate-scale bioreactors in academic and industrial institutions to produce cells for various applications in drug screening and/or cell therapy. However, the application of these bioreactors in cultivating hiPSC-derived immune cells and other blood cells is noticeably lacking. To address this gap, we have developed a xeno-free and chemically defined intermediate-scale bioreactor platform, which allows for the generation of standardized human iPSC-derived hematopoietic organoids and subsequent continuous production of macrophages (iPSC-Mac).

**Methods:**

We describe a novel method for intermediate-scale immune cell manufacturing, specifically the continuous production of functionally and phenotypically relevant macrophages that are harvested on weekly basis for multiple weeks.

**Results:**

The continuous production of standardized human iPSC-derived macrophages (iPSC-Mac) from 3D hematopoietic organoids also termed hemanoids, is demonstrated. The hemanoids exhibit successive stage-specific embryonic development, recapitulating embryonic hematopoiesis. iPSC-Mac were efficiently and continuously produced from three different iPSC lines and exhibited a consistent and reproducible phenotype, as well as classical functionality and the ability to adapt towards pro- and anti-inflammatory activation stages. Single-cell transcriptomic analysis revealed high macrophage purity. Additionally, we show the ability to use the produced iPSC-Mac as a model for testing immunomodulatory drugs, exemplified by dexamethasone.

**Conclusions:**

The novel method demonstrates an easy-to-use intermediate-scale bioreactor platform that produces prime macrophages from human iPSCs. These macrophages are functionally active and require no downstream maturation steps, rendering them highly desirable for both therapeutic and non-therapeutic applications.

**Supplementary Information:**

The online version contains supplementary material available at 10.1186/s13287-024-03785-2.

## Background

Human induced pluripotent stem cells (hiPSCs) have been widely used in therapeutic studies, including cell therapy and tissue engineering [1–3], as well as in non-therapeutic applications such as drug discovery, disease modeling and developmental studies [4–6]. Of note, both therapeutic and non-therapeutic applications require varying amounts of iPSC-derived cells, depending on the specific application, and the current up-scaling to therapeutic scales may not be required for non-therapeutic in vitro studies. This necessitates the development of methods to produce iPSC-derived cells in vessels of intermediate scale.

Multiple reports have shown that drug candidates have a high failure rate of 90% or higher due to various reasons [7, 8]. Moreover, the effectiveness of several drug candidates, initially screened in mouse or larger animal models, later proved to be ineffective in the treatment of human patients [9]. This underscores the need for more physiological test systems, such as hiPSC-derived models to accurately recapitulate the disease and ultimately develop successful drug candidates or therapies [10–12]. For instance, patient-derived hiPSC-neurons have been shown to be valuable in validating drugs in the context of familial dysautonomia and Rett syndrome [13, 14]. Additionally, with several promising drug candidates undergoing testing for specific diseases, reports have linked them to immunotoxicity. This emphasizes the importance of validating drugs within the context of the immune system [15–17].

Hematopoietic stem cells and immune cells have gained enormous interest in therapeutic and non-therapeutic applications [18]. Thus, much effort has been dedicated to generating blood cell types from iPSCs, and substantial progress has been made in establishing robust protocols to generate iPSC-derived immune cells like macrophages, natural killer (NK) cells, dendritic cells, T cells, granulocytes and others [19–24]. One important immune cell type of interest is macrophages. Macrophages have emerged as a promising immune cell for drug screening and cell therapy applications [25, 26]. This is particularly attributed to their crucial and diverse roles as guardians of the immune system but also of tissue homeostasis. Macrophages are specialized phagocytes involved in the clearance of pathogens, debris and tissue homeostasis [27]. Moreover, macrophages play a key role in modulating the immune system by initiating pro-inflammatory or anti-inflammatory responses, thus, contributing to a plethora of diseases such as cancer, chronic infections, fibrosis or autoimmune diseases [28]. Cutting-edge immunotherapeutic approaches aim to rectify disease-promoting disparities between pro- or anti-inflammatory activation of macrophages. Nonetheless, the utilization of traditional cell models like cancer cell lines and primary monocyte-derived macrophages for drug screening focused on addressing aberrant macrophage activation is hindered by constraints such as diminished physiological relevance, restricted cell accessibility or donor-to-donor variabilities.

The current protocols for the production of immune cells use two main approaches: 2D planar and 3D dynamic cell culture systems. Considering the imperative for large-scale cell production, utilizing suspension-based, scalable cell cultures present a much more advantageous approach than simply scaling-out adherent systems. At the moment, the majority of the manufacturing in most academic and non-academic settings involves the use of 2D planar vessels as open culture systems, which result in batch-to-batch variability, high manufacturing costs, and labor-intensive operations that are prone to human manufacturing errors. Thus, there is a substantial demand for intermediate-scale cell production in academic and industrial settings. This is particularly crucial for early stages of cell manufacturing process development and applications in early drug discovery, positioning bioreactor systems as an ideal choice for intermediate-scale cell manufacturing. Currently, there are limited options available for semi-closed intermediate-scale culturing systems. For instance, the benchtop bioreactors available on the market facilitate the monitoring and control of process parameters including temperature, pH and CO_2_ levels. Different versions show the capability to simultaneously culture multiple reactor vessels, each with a maximum volume of 15–50 ml. Currently, benchtop bioreactors have been employed for expanding iPSCs, and differentiating various cell types such as adipose cells, cardiac, neuronal, epithelial cells and hepatocytes [29–34]. However, its application in cultivating hiPSC-derived immune cells and other blood cells remains conspicuously absent. Considering the vital role of immune cells in both therapeutic and non-therapeutic applications and recognizing the substantial demand for intermediate-scale processes, this represents a significant gap in current utilization.

The objective of this study is to provoke the development of a standardized and fully-defined intermediate-scale bioreactor platform to produce hiPSC-derived hematopoietic organoids and macrophages, building on our expertise in the continuous production of hiPSC-derived immune cells [20, 23, 35, 36]. To achieve this goal, we utilized an intermediate-scale benchtop bioreactor platform and demonstrated the continuous generation of viable, characteristic and functional hiPSC-derived hematopoietic organoids and macrophages (iPSC-Mac) from three different hiPSC lines. We characterized the iPSC-Mac from different lines and harvests, comparing phenotype and function to primary macrophages derived from peripheral blood monocytes. Importantly, we also demonstrated the applicability of iPSC-Mac for drug testing using the well-established anti-inflammatory agent dexamethasone.

## Methods

### Human pluripotent stem cell cultivation

Experiments were performed utilizing three different human induced pluripotent stem cell (hiPSC) lines: hiPSC line#1: hCD34iPSC11 (https://hpscreg.eu/cell-line/MHHi015-B [37]), hiPSC line#2: LiPSC-GR1.1 (https://hpscreg.eu/cell-line/RUCDRi002-A [38]) and hiPSC line#3: hHSC_Iso4_ADCF_SeViPS2 (https://hpscreg.eu/cell-line/MHHi001-A [39]. Prior to process inoculation, hiPSCs were expanded in feeder-free monolayer culture on Geltrex™—or vitronectin coated plates (both ThermoFisher Scientific). hiPSCs were cultured in an incubator at 37 °C with 5% CO2, the detailed cultivation of hiPSCs can be found in supplementary methods.

### Hematopoietic differentiation of hiPSC to macrophages with GMP-compatible upscaling platform

For the described study, the 3D Cell Culture Incubator CERO 3D (OLS OMNI Life Science) was used for the 3D generation of hematopoietic organoids “hemanoids” to produce iPSC-derived macrophages (iPSC-Macs). Three hiPSC lines were used as starting material. As a first step, 3D aggregates were generated in suspension culture and mesoderm priming was used to induce the hematopoietic specification in the organoids. Here, 3 × 10^6^ single cell hiPSCs were inoculated into 18 ml mesoderm priming medium 1a (E8 medium + hVEGF 50 ng/ml, hBMP-4 50 ng/ml, hSCF 20 ng/ml (all Peprotech) and 10 mM Y-27632) using a customized program for this step (program entails: rotation speed 80 rpm and rotation period 2 s for 26 h). On day 1, after 26 h, tiny aggregates were observed and the medium was exchanged via centrifugation of the aggregates (200× *g* for 1 min) and addition of 18 ml mesoderm priming medium 1b (E6 medium + hVEGF 50 ng/ml, hBMP-4 50 ng/ml, hSCF 20 ng/ml and 10 mM Y-27632). At this stage the rotation speed of the reactor was reduced to 65 rpm with a rotation period of 4 s. On day 2, the volume was increased to 36 ml by the addition of 18 ml of mesoderm priming medium 1b and rotation speed was adjusted to 75 rpm with a rotation period of 4 s. On day 4, a full medium exchange was performed by allowing the formed aggregates to sediment by gravity and addition of 40 ml mesoderm priming medium 2 (E6 medium + hVEGF 50 ng/ml, hBMP-4 50 ng/ml, hSCF 20 ng/ml and hIL-3 25 ng/ml). The rotation speed was adjusted to 80 rpm with a rotation period of 4 s. A full medium exchange was performed on day 7 by allowing the aggregates to sediment and followed by the addition mesoderm priming medium 2. On day 10, the formed aggregates were ready for hematopoietic differentiation towards macrophage fate, which was initiated by a full medium exchange of 40–45 ml macrophage differentiation medium (X-VIVO 15 (Lonza) + 1% penicillin–streptomycin (Gibco) + 2 mM L-glutamine (Invitrogen) + 0.05 mM b-mercaptoethanol (Invitrogen) + hIL-3 25 ng/ml and hM-CSF 50 ng/ml (both Peprotech). The rotation speed remained 80 rpm and rotation period was adjusted of 2 s. Subsequent medium changes—equaling to macrophage harvests—were performed by allowing the hemanoids to sediment by gravity, followed by removal of the medium containing produced iPSC-Macs and the addition of fresh macrophage differentiation medium. Medium changes/harvests were performed twice in the first week and subsequently every 7 days for the continuous production of macrophages over the span of multiple weeks. Harvested iPSC-Macs were filtered through a 70 µm filter (Pluriselect) before further analysis/experiments.

### Isolation and differentiation of human monocytes to macrophages from peripheral blood mononuclear cells

Human buffy coat blood was provided by ‘Deutsches Rotes Kreuz Niedersachsen’, with the prior informed consent of all respective donors. Two steps of density gradient centrifugation were followed to isolate monocytes from the buffy coat samples as described previously [35], for more details see also supplementary methods file. Isolated monocytes were cultured for 10–14 days in RPMI 1640 medium (ThermoFisher Scientific) supplemented with 10% fetal calf serum (FCS), 2 mM L-glutamine, 1% penicillin–streptomycin, with the addition of 25 ng/mL hM-CSF and 12.5 ng/mL hIL-3 for 5 days. Following that, medium was changed to the same RPMI medium but with only 25 ng/mL hM-CSF for an additional period of 7–10 days. After that, the primary monocytes-derived macrophages (MDMs) were collected using Trypsin (Invitrogen) and used in the different assays.

### Phagocytosis assay using pHrodo™ BioParticles™

To evaluate the phagocytic capacity of the different macrophages, 2.5 × 10^5^ cells per well were seeded into a 12-well culture plate in iPSC-Mac terminal differentiation medium (X-VIVO 15 medium + 1% penicillin–streptomycin + 2 mM L-glutamine + 0.05 mM b-mercaptoethanol + hM-CSF 50 ng/ml). For this specific assay, TheraPEAK™ X-VIVO™ 15 was used (TheraPEAK™ X-VIVO™ 15, Lonza,). Seeded cells were incubated with 10 µl of pHrodo™ Red E. coli BioParticles™ (#P35361; ThermoFisher Scientific). After 2 h, the cells were collected from the wells and the phagocytosis rate was analyzed using flow cytometry (CytoflexS, Beckman Coulter). Samples without particles served as controls. The specific pHrodo™ Red dye applied in this assay only shows a fluorescent signal at low pH values in the phagolysosome, and thus can be used to discriminate true phagocytic events from particles attaching to the cell surface.

### Measurement of reactive oxygen species (ROS assay)

To evaluate the production of ROS from the receptive macrophages, DHR (Dihydrorhodamine 123, Invitrogen) and PMA (phorbol myristate acetate, Sigma-Aldrich) were used. Different macrophages (iPSC-Macs/ MDMs) were treated with three different conditions, each condition used 2 × 10^5^ cells and the conditions were as follows: unstained (no DHR and no PMA), stained unstimulated (DHR), stained stimulated (DHR + PMA). To prepare the cells for the staining and/or the stimulation, the cells were resuspended in 500 µl HBSS solution with Ca^2+^/Mg^2+^ (Invitrogen) and incubated at 37 °C for 5 min at 175 rpm on a shaker. Equal amounts of the cell solution (2 × 10^5^ cells) were transferred to the respective tubes. Starting with the stained stimulated condition, PMA (20 μg/ml final concentration) was added to stimulate the cells and incubated at 37 °C for 5 min at 175 rpm. Next, DHR (10 μg/ml final concentration) was added to the following tubes: stained stimulated and stained unstimulated and incubated at 37 °C for 15 min on a shaker. Subsequently, tubes were transferred on ice and incubated for 30 min. Lastly, the samples were evaluated for their ROS production by measuring the oxidized rhodamine dye using flow cytometry (CytoflexS, Beckman Coulter).

### Cytokine secretion after LPS stimulation (ELISA)

The respective macrophages generated from the different hiPSC lines or from PBMCs were seeded at 2.5 × 10^5^ cells per well of a 12 well-culture plate in iPSC-Mac terminal differentiation medium and either left unstimulated or were stimulated with 500 ng/ml of LPS (Sigma). 4 h post-treatment the supernatants were collected and stored at − 80 °C for later cytokine analysis. Human IL-6 concentrations were determined from the frozen supernatants using the DuoSet ELISA Kit (R&D Systems) following the manufacturer’s instructions.

### Polarization of human iPSC macrophages towards different activation status

Human iPSC-Macs were seeded in multi-well plates (4 × 10^5^ cells per well of a 12-well plate or 1 × 10^6^ cells per well of a 6-well plate) and cultured in iPSC-Mac terminal differentiation medium (X-VIVO 15 medium + 1% penicillin–streptomycin + 2 mM L-glutamine + 0.05 mM b-mercaptoethanol without cytokine addition.) for three to five days of terminal differentiation. Subsequently, cells were stimulated with 25 ng/ml hIFNy, 10 ng/ml, hIL-4 or 10 ng/ml, hTGFb1 and hIL-10 (all Peprotech) in iPSC-Mac terminal differentiation medium for 24 h (using 100 µl medium per 1 × 10^5^ cells). Control cells were only cultured in iPSC-Mac terminal differentiation medium. After 24 h, supernatants were collected and frozen at − 80 °C and the cells were harvested and analyzed by flow cytometry (CytoflexS).

### ***Cytokine secretion after polarization (LEGENDplex***.***™)***

The LEGENDplex™ Human M1/M2 Macrophage Panel (10-plex) kit (BioLegend) was used to quantify levels of secreted cytokines following the manufacturer’s instructions. Stained samples were measured by flow cytometry on a CytoflexS and analyzed with the LEGENDplex™ software provided by BioLegend.

### Cytospin staining

For cytospin staining, 2 × 10^4^ or 3 × 10^4^ cells were centrifuged on glass slides using a Cytofuge® (Medite) at 700 RPM for 10 min. The slides were left to air-dry, and were stained with 0.25% (w/v) of May-Grünwald solution (Roth) for 5 min and washed three times with distilled water. The second staining round was performed using 5% GIEMSA solution (Roth) for 20 min. Afterwards the slides were thoroughly washed three times with distilled water. Stained and air-dried cells were fixed with ROTI^®^Histokitt mounting solution (Roth) and imaged at a Olympus BX41 microscope.

### Flow cytometry

Flow cytometry analysis was performed on the different macrophage populations and hemanoids. The hemanoids were dissociated to single cells by TrypLE™ Express treatment (ThermoFisher Scientific) for 7–10 min at 37 °C. During the dissociation, pipetting was performed to facilitate the dissociation of the aggregates. A viability dye (Zombie Aqua fixable viability kit, BioLegend) was used following the manufacturer’s recommendation. FACS buffer (PBS + 2 mM EDTA + 5% FCS) was used for cell staining and FcR blocking reagent (1:100, Miltenyi #130-059-901) was added prior to antibody staining. Staining was performed on ice for 30 min in the dark with respective antibody panel (detailed information on the used antibodies and dilutions can be found in the supplementary methods file). Cells were washed and acquired with FACS buffer. Acquisition was performed by flow cytometry using the CytoflexS (Beckman Coulter). Single stained sample controls (hemanoid panel) and compensation beads (macrophage panel) were used for the compensation for multicolor staining. Analysis of the data was performed using FlowJo software (BD Bioscience).

### Histology and immunohistochemistry staining

For histologic and immunohistologic stainings, hemanoids were fixed in 4% paraformaldehyde (PFA) overnight at 4 °C, washed with PBS and stored in PBS until dehydration in a graded ethanol series and subsequently embedded in paraffin. Paraffinized samples were sliced into 3 µm sections with an RM 2265 microtome (Leica), rehydrated and stained with hematoxylin and eosin for histologic analyses. Prior to immunohistochemical analyses sections were rehydrated and underwent antigen retrieval (Dako). The samples were then blocked in 1% BSA in PBS and incubated with anti-CD45 (Stem Cell #60018), diluted 1:1000, anti-VE-cadherin (Invitrogen PA5-19612) or anti-VEGFR2 (Cell Signaling mAb #9698) in blocking solution. Detection of the primary antibody was achieved by incubation with peroxidase conjugated ImmPRESS anti-Rabbit IgG Reagent (Vector MP-7451-15) or ImmPRESS anti-Mouse (Vector MP-7402–15) and subsequent staining with DAB + substrate (Agilent #K346711-2). Counterstaining of nuclei was performed with hematoxylin.

### Quantitative reverse-transcriptase PCR

For quantitative reverse-transcription PCR (qRT-PCR), the total RNA of 0.5–1 × 10^6^ cells was isolated and subjected to DNase I digestion using the Direct-zol RNA MicroPrep Kit (Zymo) following the manufacturer´s instructions. Up to 2 µg RNA was reverse transcribed into cDNA using the RevertAid First Strand cDNA Synthesis Kit (ThermoFisher Scientific). Subsequently, qRT-PCR assays were performed with 10 ng input cDNA on a 7500 Fast Real-Time PCR thermocycler (Applied Biosystems) with 40 cycles of 95 °C for 15 s and 60 °C for 1 min. TaqMan-based qRT-PCR was performed using TaqMan Universal Master Mix II, UNG (Applied Biosystems) with the following pre-designed probes obtained from Applied Biosystems: *GAPDH* (Hs030051111_g1), *POU5F1* (Hs030051111_g1), *PTPRC* (Hs00365634_g1), and *SOX17* (Hs00751752_s1). SYBR Green-based assays were performed using SYBR Green PCR Master Mix (Applied Biosystems) with pre-designed Quantitect Primer Assays (Qiagen) for *GAPDH* (QT00079247), *KDR* (QT00069818), and *RUNX1* (QT00026712). Respective gene expression is shown relative to *GAPDH* expression.

### Single-cell RNA-seq

Human iPSC-Mac derived from the different iPSC lines were harvested as described before. Viable cells were purified by fluorescence activated cell sorting (FACS) on basis of scatter properties and exclusion of 4′,6-diamidino-2-phenylindole (DAPI) positive events, which has been added immediately before sorting (0.1 µg/ml final concentration). Library generation was performed according to manufactures recommendations. Additional information regarding sequencing, processing, and analysis of the sample can be found in supplementary methods.

### In vitro screening assay for anti-inflammatory drugs employing dexamethasone with concurrent pro-inflammatory stimulation

To establish a setup in which the respective macrophages can be screened for the activity of anti-inflammatory agents, we conducted a screening assay employing Dexamethasone as the appropriate positive control for such drugs. In the assay, 5 × 10^4^ of the respective iPSC-Macs or MDMs were seeded per well in 96-well plates cultured in a volume of 200 μl/well with iPSC-Mac terminal differentiation medium for three to five days of terminal differentiation. The terminally differentiated cells were then stimulated with increasing concentrations (1–500 ng/ml) of LPS (Sigma-Aldrich), treated with or without concurrent administration of Dexamethasone at 1 μg/mL (Sigma-Aldrich). After 4 h of stimulation, the supernatants were collected and frozen at − 20 °C for further quantification of hIL-6 secretion using an ELISA assay.

### Statistical analysis

Statistical analysis was performed using Prism version 9 (GraphPad). The type of analysis of variance (ANOVA) as well as Post-hoc testing is indicated in the respective figure legends.

## Results

### Organoid-based production of iPSC-Mac in intermediate scale bioreactors recapitulates embryonic hematopoietic development

Based on our previous work demonstrating the efficient production of iPSC-derived macrophages [20, 23, 36], we here introduce a standardized, xeno-free, chemically defined and easy-to-use intermediate-scale benchtop bioreactor platform dedicated to producing iPSC-derived macrophages via 3D hematopoietic organoids, which we refer to as hemanoids.

**Fig. 1:**
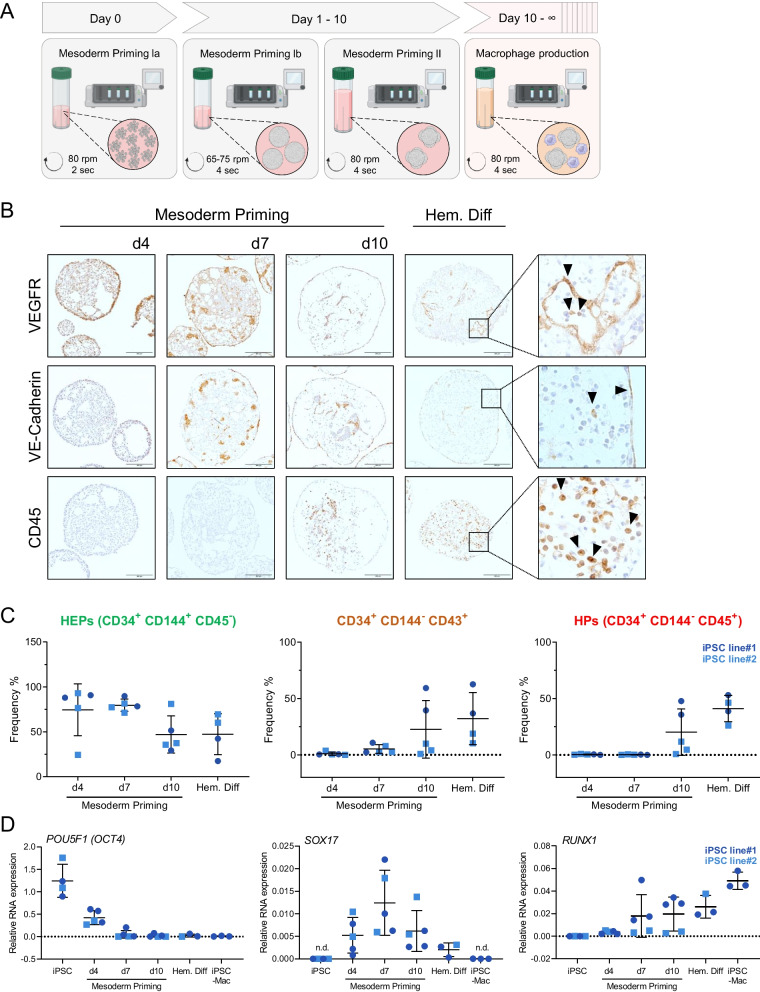
Organoid-based production of iPSC-Mac in intermediate-scale bioreactors recapitulates embryonic hematopoietic development. **A** Schematic representation of the manufacturing process in an intermediate-scale benchtop bioreactor to produce hemanoids and the continuous generation of iPSC-derived macrophages.** B** Immunohistochemical analysis of VE-Cadherin/CD144, VEGFR and CD45 expression in hemanoids derived from day 4, 7 and 10 of mesoderm priming as well as from hemanoids during hematopoietic differentiation after they initiated production of iPSC-derived Mac (day 7–10). Arrows indicate characteristic regions (scale bar = 200 µm, data shown for iPSC line#1, representative of n = 2).** C** Flow cytometric analysis of CD34, CD144, CD43 and CD45 expression to identify different hemato-endothelial progenitor populations. Hemanoids were dissociated and analyzed on day 4, 7 and 10 of mesoderm priming as well as during hematopoietic differentiation after they initiated production of hiPSC-derived Mac. Populations were pre-gated for single cells, CD34^+^ cells and viable cells. Subsequently, the frequency of CD144^+^/CD45^−^ Hemato-endothelial progenitors, CD144-/CD43^+^ early hematopoietic progenitors and CD144-/CD45^+^ hematopoietic progenitors was analyzed in the CD34^+^ population (individual values with mean ± SD, iPSC line#1: dark blue dots, n = 2 and iPSC line#2: blue squares, n = 3. Gating strategy and representative plots can be found in Supplementary Fig. S1). **D** Gene expression analysis of key genes for pluripotency (*POU5F1* (OCT4)), hemato-endothelial progenitors (*SOX17*), and hematopoietic progenitors (*RUNX1*) at different stages of differentiation as well as in hiPSC-derived macrophages (iPSC-Mac) by qRT-PCR. Values are represented as relative RNA expression to *GAPDH* (housekeeping gene) (individual values with mean ± SD, iPSC line#1: dark blue dots, and iPSC line#2: blue squares, n = 2–3 per line, n.d. indicates detection limit of the target gene)

To generate hemanoids, we initiated the differentiation process in a benchtop bioreactor by introducing a single-cell solution of hiPSCs along with mesoderm priming cytokines (SCF, BMP4, VEGF) in defined E8 medium for 24 h to allow for aggregate formation. Subsequently, the medium was changed to mesoderm priming medium 1b, and the aggregates were cultivated for an additional 3 days. On days 4 and 7, we performed another medium change for the developing hemanoids and added IL-3 along with the mesoderm priming cytokines. After 10 days of mesoderm priming, the medium was changed to hematopoietic differentiation medium containing only IL-3 and M-CSF, and the culture of the hemanoids was continued for several weeks for macrophage production (Fig. 1A).

To elucidate the developmental processes and hematopoietic commitment in the hemanoids during the mesoderm induction in a benchtop bioreactor, we analyzed the hemanoids derived from two iPSC lines: (our in-house, lenti-viral reprogrammed hiPSC line (hCD34iPSC11, iPSC line#1) [37] and an episomal reprogrammed, potential GMP-compatible hiPSC line (LiPSC-GR1.1, iPSC line#2) [38] at days 4, 7, 10 of mesoderm priming and after one week of hematopoietic differentiation, when the hemanoids began to produce iPSC-derived macrophages. In more detail, we dissected the cellular composition of our hemanoids and performed immunohistochemistry, immunophenotyping, and gene expression to determine the emergence of crucial markers and cell populations during the successive development of the hemanoids.

The early mesoderm marker VEGFR2/KDR displayed prominent expression at day 4 with gradual decrease at day 7 and pronounced reduction at day 10 and during hematopoietic differentiation as shown by immunohistochemistry and RT-qPCR (Fig. 1B, Fig. S1A). Additionally, we observed VE-Cadherin/CD144 expression (indicating the development of (hemato-endothelial cells) at low levels already at day 4, a clear increase at day 7 and gradually decrease at day 10 and during hematopoietic differentiation. Expression of CD45, demonstrating the emergence of hematopoietic cells was observed for the first time at day 10 and during hematopoietic differentiation (Fig. 1B, Fig. S1B–D).

To delineate the successive development and emergence of hemato-endothelial progenitor cells (HEPs) CD34^+^/CD144^+^/CD45^−^, CD34^+^/CD144^−^/CD43^+^ cells and hematopoietic progenitors (HPs) CD34^+^/CD144^−^/CD45^+^ during hemanoid formation, we dissociated the hemanoids and analyzed the cells by flow cytometry. Among the CD34^+^ cells, HEPs were first detected at day 4 and 7 in our early hemanoids (74.5 ± 25.4%, 79.7 ± 6.0%, mean ± SD, n = 5, respectively), and a clear decrease at day 10 followed by gradual reduction during hematopoietic differentiation (47.0 ± 18.5%, mean ± SD, n = 5 and 31.8 ± 20.9%, mean ± SD, n = 4, respectively) was noted. Furthermore, we observed diminutive expression of CD34^+^/CD144^−^/CD43^+^ during earliest stage day 4 (1.0 ± 1.3%, mean ± SD, n = 5). The first appearance of HPs, more specifically, CD144^−^/CD43^+^ cells among the CD34^+^ cells was observed at day 7 and showed a gradual increase at day 10 and during hematopoietic differentiation (5.2 ± 3.4%, mean ± SD, n = 5 and 22.0 ± 22.8%, mean ± SD, n = 5, respectively). Interestingly, we observed CD34^+^/CD144^−^/CD45^+^ HPs at later stage than early CD34^+^/CD144^−^/CD43^+^ cells. The first appearance of CD34^+^/CD144^−^/CD45^+^ cells was observed at day 10 (20.2 ± 18.0%, mean ± SD, n = 5) and increased during further hematopoietic differentiation (4.1 ± 10.1%, mean ± SD, n = 4) (Fig. 1C, Fig. S1B).

Quantitative gene expression analysis of the entire hemanoids confirms a substantial early decrease in pluripotent marker *OCT4*, which subsequently decreased further at later stages. Transient expression *SOX17*, a transcription marker expressed in hemogenic endothelial cells, was noted in hemanoids at day 4–10. Additionally, expression of *RUNX1* a marker required for the emergence of HPs during embryonic development and lymphoid/myeloid lineage maturation, appeared at day 7 and progressed during hemato-myeloid differentiation (Fig. 1D).

Taken together, we demonstrate that hiPSC-derived hemanoids undergo a stage-specific successive development, recapitulating key aspects of human embryonic hematopoiesis.

### Phenotypic characterization of iPSC-Mac continuously produced in intermediate-scale bioreactors

**Fig. 2:**
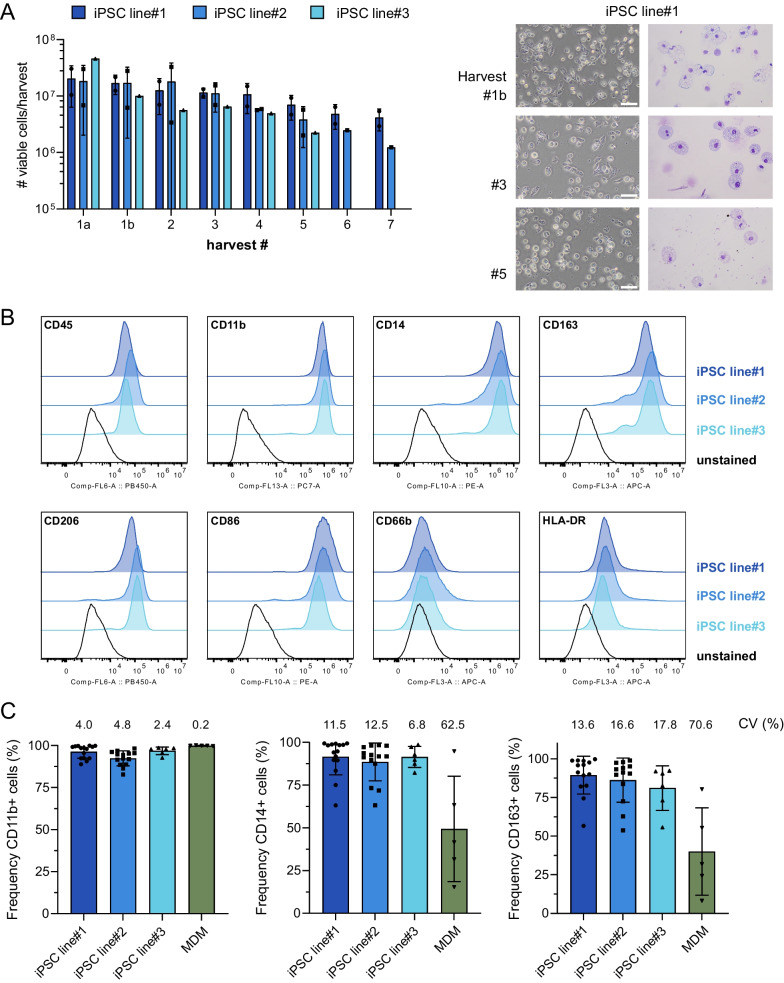
Phenotypic characterization of iPSC-derived macrophages continuously produced in intermediate scale bioreactors. **A** Number of viable cells harvested from the intermediate scale bioreactor for three different iPSC lines (iPSC line#1: n = 2, iPSC line#2: n = 2, iPSC line#3: n = 1, all individual values with mean +/− SD) over a time span of 7 weeks. Representative brightfield images and cytospin staining for iPSC-derived macrophages (iPSC-Mac) derived from harvest #1b, 3 and 5 for iPSC line#1. **B** Representative flow cytometry analysis of CD45, CD11b, CD14, CD163, CD206, CD86, HLA-DR and CD66b expression on iPSC-Mac from harvest #5. Histograms represent unstained iPSC-Mac (black line), and stained iPSC-Mac derived from iPSC line#1 (dark blue filled), iPSC line#2 (blue filled) and iPSC line#3 (light blue filled). Cells were pre-gated for viable cells according to FSC/SSC properties as well as single cells using SSC-A/SSC-H (see Fig S2B for gating strategy). **C** Frequency of CD11b^+^, CD14^+^ and CD163^+^ cells derived from different harvests/differentiations of the three different hiPSC lines as well as primary monocyte-derived macrophages. Individual values with mean ± SD, n = 5–15). Coefficient of variation (CV) is given for all values

To test our differentiation process for robustness and efficiency of cell production, we subjected three different hiPSC lines to the recently developed, xeno-free, fully-defined and scalable protocol. After the formation of hemanoids in suspension culture by mesoderm priming, we observed the first production of macrophage-like cells released into the medium after approx. 10–14 days of cultivation in hematopoietic differentiation medium supplemented with IL-3 and M-CSF. In the first week of production, cells were harvested twice (harvest 1a and 1b), whereas in the consecutive weeks, cell harvest was performed along the media change on a weekly basis. Hemanoids derived from all three human iPSC lines continuously produced iPSC-Mac for 5–7 weeks (Fig. 2A) with comparable efficiencies of around 12 Million cells on average per harvest (iPSC line#1: 11.1 ± 9.6 Million cells, iPSC line#2: 11.9 ± 7.7 Million cells, iPSC line#3: 12.6 ± 16.6 Million cells, all mean ± SD, n = 14, 12 and 6 respectively). Importantly, harvested cells showed a classical and reproducible macrophage phenotype in brightfield as well as May-Grünwald/Giemsa stained cytospin images across the different harvests and the three hiPSC-lines (Fig. 2A, Fig. S2A). Additionally, immunophenotype analysis confirmed homogenous and characteristic surface expression of CD45 and CD11b (hematopoietic/myeloid), in addition to CD14, CD163, CD206 and CD86 (monocyte/macrophages) on iPSC-Mac derived from all three tested hiPSC lines, while CD66b, as a surface marker characteristic for granulocytes was absent (Fig. 2B, C, Fig. S2B, C). In this respect, iPSC-Mac from the consecutive weeks of differentiation showed a reproducible surface marker profile. Of note, we observed a lower expression of CD14 and more pronounced in CD163 positive cells in the first week of differentiation in comparison to later stages of differentiation (Fig. S2C).

When comparing our iPSC-Mac to primary macrophages derived from peripheral blood monocytes (MDM), we observed a much more homogenous and reproducible expression of CD14 and CD163, as important markers for macrophage differentiation on the hiPSC-derived cells (Fig. 2C). While CD11b and CD86 were also homogenously expressed on primary macrophages derived from different donors (Fig. S3), we observed a strong variability of CD14 and CD163 expression between the different donors/differentiations for primary cells, which is also indicated by a large coefficient of variation (CV) of 62.5% for CD14 and 70.6% CD163 expression compared to much lower CV values of 6.8–17.8% for iPSC-Mac derived from the different lines (Fig. 2C). Interestingly, we noted differences in the expression levels of the macrophage mannose receptor CD206 as well as HLA-DR between hiPSC-derived and primary macrophages. Here, our iPSC-Mac displayed lower expression of HLA-DR, but higher levels of CD206 expression, indicating a more anti-inflammatory phenotype upon harvesting (Fig. S2C).

### Single cell transcriptomic analysis of iPSC-Mac from different hiPSC lines

To better define the iPSC-Mac and analyze the homogeneity of the population on a transcriptional level, we performed a scRNA sequencing analysis with iPSC-Mac from all three different lines, which were harvested at the same day from parallel running differentiations (all harvest #5).

**Fig. 3:**
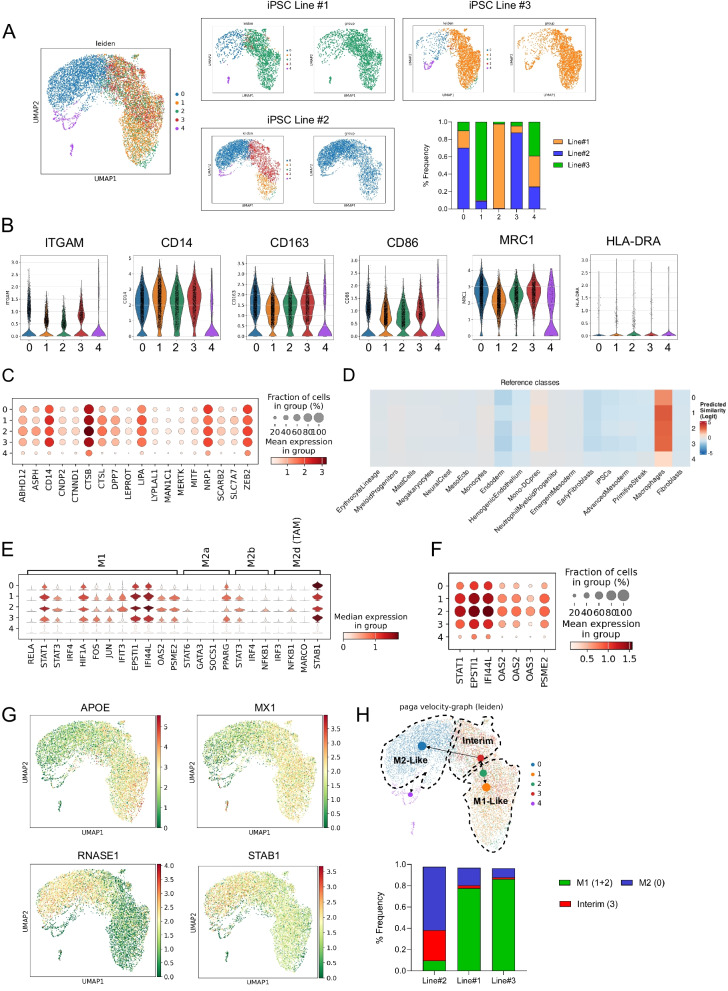
Single Cell transcriptomic analysis of iPSC-Mac from different hiPSC lines. **A** Uniform Manifold Approximation and Projection (UMAP) representing unsupervised clustering overlaying hiPSC lines #1, #2, and #3. Additional UMAP representation for each iPSC line. Bar chart representation demonstrating the ratios of each cluster in each line independently. **B** Violin plots demonstrating expression of ITGAM, CD14, CD163, CD86, MRC1/CD206, HLA-DRA for each cluster. **C** Expression of conserved lineage markers specific to macrophages (adapted from [40]) within the different clusters. **D** Heatmap representing the predicted probabilities of cell types. Cluster annotations were predicted using logistic regression classifiers trained on publicly available data [56]. **E** Gene list of macrophage polarization state M1, M2a, M2b and M2d (adapted from [57]). **F** Gene list of IFN_γ_ fingerprint (adapted from [52]). **G** UMAP demonstrating global expression of pro and anti-inflammatory genes (APOE, MX1, RNASE1, STAB1) **H** Paga plot velocity graph annotate clusters M1, interim, and M2 cell states of macrophages. Bar chart representation demonstrating the ratios of M1, M2 and interim population produced from each line independently

After combination of the three sample sets and dimensionality reduction, we observed 5 different clusters in the Uniform Manifold Approximation and Projection (UMAP) analysis. Importantly, all iPSC-Mac samples from the different iPSC lines show cells belonging to all 5 clusters, though with variable frequencies. Here, cells allocated to cluster 0 and 3 were most prominently in the iPSC-Mac derived from iPSC-line#2, whereas cells allocated to cluster 2 were mainly found in iPSC-Mac derived from iPSC line#1 and cells allocated to cluster 1 were derived from iPSC-Mac derived from iPSC line#3 (Fig. 3A). Irrespectively of these differences, we observed, that clusters 0–3 all demonstrate a clear macrophage phenotype indicated by the expression of ITGAM/CD11b, CD14, CD163, CD86, and MRC1/CD206, as also previously observed by flow cytometry. Also, the absence/very low expression of HLA-DR was confirmed. Here, only cells allocated to the small cluster 4 showed absence of these important macrophage genes, indicating some few contaminating cells (Fig. 3A, B). Similar results were obtained, when analyzing typical macrophage genes (conserved macrophage genes [40]). Also, here we noticed a strong expression of these key genes over clusters 0–3, whereas low to no expression was detected in cluster 4 (Fig. 3C). To confirm the cell identity, we performed logistic regression (LR) analysis using publicly available single cell transcriptomics data. Strikingly, we observed expression related to the class reference of macrophages and residual expression related to mono-DC precursor and monocytes. Within the macrophage reference class, the highest expression was observed in clusters 1, 2, 3, 0 and 4, respectively (Fig. 3D). Given the primitive fingerprint of several iPSC-derived progeny, which has also been reported for iPSC-derived macrophages [41, 42], we also analyzed the expression of genes associated with primitive yolk sac macrophages [36]. Indeed, we observed expression of LYVE1, NID1, LIN28B, IGF2BP1, CALD1, FGF13, SERPINH1, FERMT2 and PARD3 in clusters 0–3 (Fig. S4). To better understand the differences in clusters 0–3, we analyzed the expression patterns of genes, which are associated with macrophage activation/polarization. Here, we observed some differences between the clusters. While all of the clusters demonstrated expression of genes associated with pro- and anti-inflammatory activation, especially cluster 1 and 2 showed a more abundant and stronger expression of genes associated with a pro-inflammatory M1 macrophages activation status such as MX1, STAT1, or HIF1A. On the other hand, STAB1, a marker gene for anti-inflammatory or primitive/tissue resident macrophages [43] as well as RNASE1, which was recently described as a M2 macrophage marker [44] was highly expressed in cluster 0 and 3 (Fig. 3E–G). However, the clusters do not reflect a black and white picture of macrophage polarization, but demonstrate co-expression of key genes associated with the both directions. This is also indicated by a strong expression of APOE in cluster 1, which has been shown to induce an anti-inflammatory macrophage phenotype [45] and may indicate an auto-regulatory feedback loop.

These data point out, that indeed most of the harvested and non-purified cells, represent a core macrophage transcriptome (cluster 0–3). However, specifically looking at the different macrophage activation stages, the clusters differ in abundance and expression level of specific genes associated with pro- and anti-inflammatory macrophage functions. Given the different distribution of cells between the clusters in iPSC-Mac derived from the different iPSC lines, we observe a trend towards a slightly more pro-inflammatory activation in iPSC-Mac derived from hiPSC line#1 and 3 (cluster 1 and 2 show a higher frequency of cells), while iPSC-Mac derived from iPSC line#2 displayed more of anti-inflammatory signature (cluster 0 and 3 show a higher frequency of cells) (Fig. 3G). Given the observation of various polarization genes signatures, our aim was to analyze the clusters using RNA velocity and trajectory analysis to identify the various cell states. Notably, we observed directionality from cluster 2 toward cluster 3, then to cluster 0. Additionally, cluster 2 exhibited movement toward cluster 1, suggesting the presence of three distinct cell states. We noted higher expression of genes related to an M1-like signature in clusters 1 and 2. Conversely, expression of genes related to M2-like signatures in clusters 0 and 3 seemed to represent a combination of M2-like genes with lower expression of M1 genes and cluster 0 with the lowest expression level (Fig. 3E–G). Annotating the cell clusters based on gene expression and trajectory analysis, we identified cluster 0 as an M2-like phenotype, cluster 1 and 2 as M1-like, and cluster 3 as an interim between M1 and M2. We observed the highest M1-like cell frequency in Line#3 (86.3%) followed by Line#1 (77.7%) and Line#2 (9.64%). M2-like and interim cell frequencies was the highest in line#2 (59.5% and 28.6%) followed by line#1 (16.5% and 2.55%) and line#3 (8.6% and 1.46%), respectively (Fig. 3H).

### Generated iPSC-Mac demonstrate important pro-inflammatory functionality

After showing broad phenotypic similarities between the iPSC-Mac derived from different harvests, differentiations, and hiPSC lines, we next aimed to characterize their functional properties. Given the important role of macrophages in cellular host defense, we analyzed their potential to phagocytose bacterial particles, to produce reactive oxygen species (ROS) and secrete Interleukin 6 (IL-6) in response to a pro-inflammatory stimulus.

**Fig. 4:**
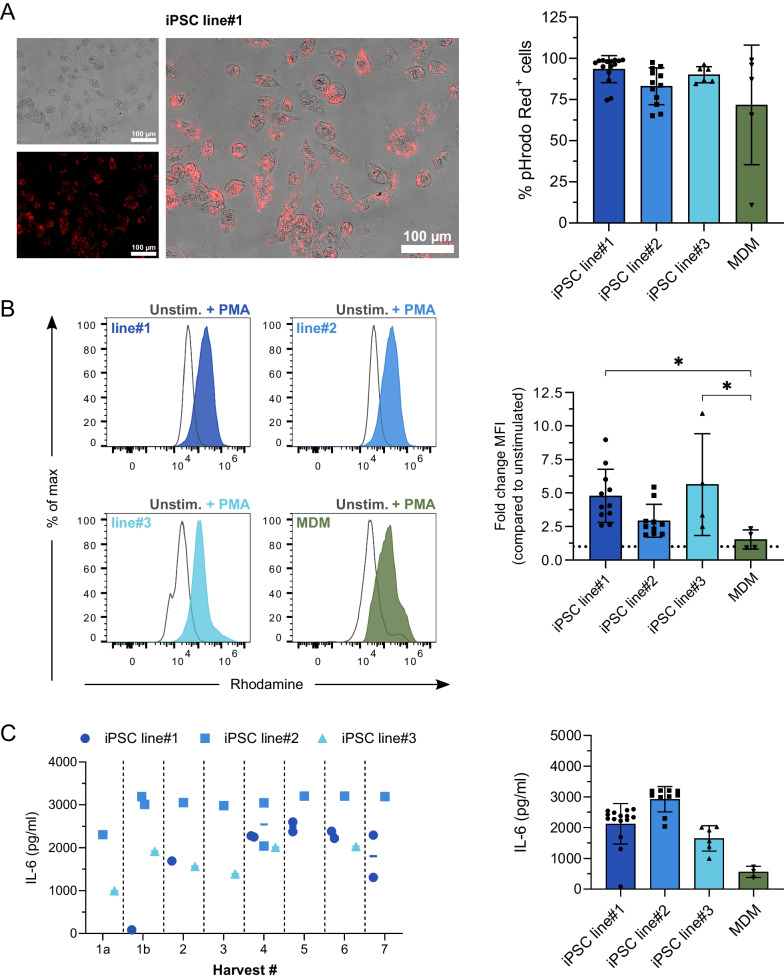
iPSC-Mac demonstrate important pro-inflammatory functionality. **A** Phagocytosis of pHrodo™ Red E. coli BioParticles. Different iPSC-Mac as well as primary Mac were incubated with pHrodo™ Red E. coli BioParticles for 2 h at 37 °C. Subsequently, phagocytosis was evaluated by the induction of a red fluorescent signal after acidification of the pH-sensitive pHrodo™ Red in the phagolysosome. Left: Representative fluorescence microscopy of iPSC-Mac derived from iPSC line#1 incubated for 2 h with pHrodo™ Red *E. coli* BioParticles (fluorescence only, brightfield as well as overlay, scale bar = 100 µm). Right: Frequency of pHrodo Red^+^ cells derived from different harvests/differentiations of the three different hiPSC lines as well as primary monocyte-derived macrophages analyzed by flow cytometry (Individual values with mean ± SD, n = 5–15). **B** Production of reactive oxygen species (ROS) by macrophages from the different sources. Different iPSC-Mac as well as monocyte derived macrophages (MDM) were incubated with PMA for 5 min and subsequently stained with Dihydrorhodamine (DHR). Left: Representative flow cytometry data for iPSC-Mac derived from the different iPSC lines#1–3 as well as primary Mac (grey: unstimulated; stained and colored filled: respective macrophages stimulated with PMA and stained). Right: Fold change of Rhodamine mean fluorescence intensity (MFI) for iPSC-Mac derived from different harvests/differentiations of the three different hiPSC lines as well as primary monocyte-derived macrophages analyzed by flow cytometry (Individual values with mean ± SD, n = 4–11, dotted line indicates “1”), **C** Secretion of IL-6 after stimulation with Lipopolysaccharide (LPS). Different iPSC-Mac as well as primary Mac were stimulated with 500 ng/ml LPS for 4 h and supernatants were analyzed for secretion of IL-6 by ELISA. Left: IL-6 levels secreted by iPSC-Mac from the three different iPSC lines for the individual harvests. Right: IL-6 secretion for iPSC-Mac derived from different harvests/differentiations of the three different hiPSC lines as well as primary monocyte-derived macrophages (Individual values with mean ± SD, n = 3–15). Statistical analysis was performed using one-way ANOVA with Tukey’s multi comparisons test (*p < 0.05, **p < 0.01, ***p < 0.001, ****p < 0.0001, ns denotes not significant)

After 2-h incubation with pHrodo™ Red *E. coli* BioParticles, we observed a strong phagocytic activity of iPSC-Mac with 93.4 ± 8.2% pHrodo™ Red positive cells for iPSC line#1, 83.0 ± 11.1% for iPSC line#2 and 90.0 ± 4.9% cells for iPSC line# 3 (n = 6–15). In contrast, we only observed a phagocytosis rate of 71.7% for MDMs, again with a strong variability indicated by a SD of 36.4% (n = 5) and a CV of 50.75% (Fig. 4A, Fig. S5A).

Stimulation with phorbol 12-myristate 13-acetate (PMA) lead to the production of ROS, indicated by an increase in Rhodamine mean fluorescence intensity (MFI) in both, iPSC-Mac and MDMs. While all different macrophages responded to PMA stimulation with an increased Rhodamine signal/ROS production, there was a general trend towards a higher induction of ROS production in the iPSC-Mac, specifically in hiPSC line#1 and 3 that showed significantly higher induction of ROS when compared to MDMs (Fig. 4B, Fig. S5B).

After stimulation with Lipopolysaccharides (LPS) for 4 h, we observed a significant induction of IL-6 secretion, indicating an efficient pro-inflammatory response of the iPSC-Mac. While we observed some differences in the level of IL-6 secreted after LPS stimulation of the iPSC-Mac derived from the different hiPSC-lines, iPSC-Mac from the different harvests of the same differentiation responded in a reproducible manner towards this stimulus. In general, we observed a higher induction of IL-6 secretion in iPSC-Mac derived from all the three lines when compared to primary macrophages (Fig. 4C).

Taken together, all iPSC-Mac displayed typical macrophage functionality and important anti-bacterial functions when compared to primary cells. Moreover, iPSC-Mac derived from the different harvests or differentiations produced on the intermediate scale, continuous production platform showed a high-quality reproducibility of phenotype and function.

### iPSC-Mac can be polarized into different pro- and anti-inflammatory activation stages

**Fig. 5:**
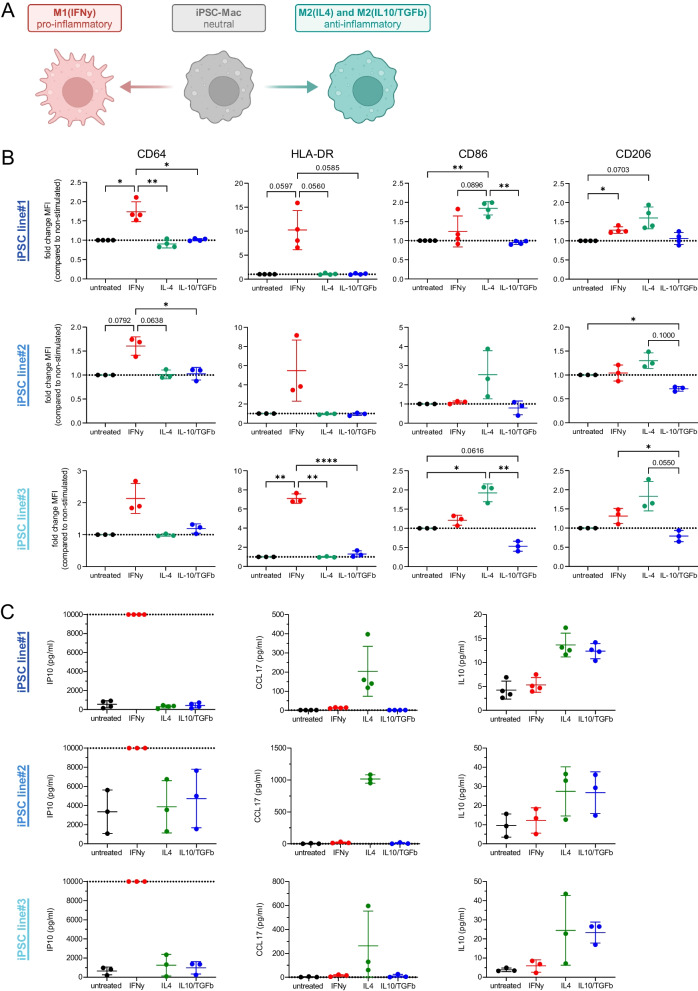
Polarization of iPSC-Mac into different pro- and anti-inflammatory activation stages. **A** Schematic representation of the experimental layout. iPSC-Mac from the three iPSC-lines were polarized in vitro by the stimulation with 25 ng/ml IFNy into pro-inflammatory M1(IFNy) macrophages or with 10 ng/mL IL-4 or IL-10/TGFb into anti-inflammatory M2(IL-4) or M2 (IL10/TGFb) iPSC-Mac. **B** Changes in surface marker expression of CD64, HLA-DR, CD86 and CD206 24 h after polarization analyzed by flow cytometry. Values are given as fold change in the median fluorescence intensity (MFI) compared to non-stimulated cells (Individual values with mean ± SD, n = 4 for iPSC line#1, n = 3 for iPSC line#2 and 3). Statistical analysis was performed using one-way ANOVA with Tukey’s multi comparisons test (*p < 0.05, **p < 0.01, ***p < 0.001, ****p < 0.0001, ns denotes not significant). **C** Secretion of IP10, CCL17 and IL10 24 h after polarization analyzed by Legendplex technology (Individual values with mean ± SD, n = 4 for iPSC line#1, n = 3 for iPSC line#2 and 3, dotted lines depict upper (IP10) or lower (CCL17) detection limits)

Given the diverse function of macrophages in innate immunity as well as tissue homeostasis and repair, a crucial feature is their responsiveness to different pro- or anti-inflammatory stimuli and a change of their activation status. Thus, we next analyzed the potential of our iPSC-Mac to adopt pro- or anti-inflammatory activation stages by stimulation with IFNy (M1), IL-4 (M2a) and IL10/TGFb (M2c) (Fig. 5A). Indeed, all iPSC-Mac derived from the three hiPSC lines responded to a pro-inflammatory IFNy stimulation with a specific up-regulation of Fc receptor CD64 as well as increased HLA-DR expression (Fig. 5B, Fig. S6, Supplementary Table 1). The anti-inflammatory stimulation with IL-4 resulted in a profound up-regulation of the T cell co-stimulatory molecule CD86 as well as the macrophage mannose receptor CD206 (Fig. 5B, Fig. S6, Supplementary Table 1), which are both associated with an anti-inflammatory activation stage. The alterations in the surface marker profile after different stimuli were associated with the induction of cytokine secretion (Fig. 5C). Here, we observed a characteristic pattern of cytokine induction across the iPSC-Mac from all hiPSC lines: IFNy induced strong induction of C-X-C motif chemokine ligand 10 (CXCL10)/ Interferon gamma-induced protein 10 (IP-10) secretion. IL-4 stimulation resulted in the specific up-regulation of Chemokine (C–C motif) ligand 12 (CCL12), and IL-4 as well as IL10/TGFb stimulation lead to the up-regulation of IL10 secretion. Whereas the pattern of cytokine induction was the same for all different lines, we observed differences in the levels of cytokine secretion. The iPSC-Mac derived from hiPSC line#1 and 3 showed only low background levels of IP10 secretion in the non-stimulated controls (558.12 ± 376.78 pg/ml, mean ± SD, n = 4 and 657.62 ± 413.87 pg/ml, mean ± SD, n = 3, respectively). In contrast, iPSC-Mac from line#2, even under steady-state conditions exhibited profound levels of IP10 secretion of 3350.21 ± 2271.28 pg/ml (mean ± SD, n = 3). Additionally, variations in CCL17 secretion levels were observed after IL-4 stimulation among the different lines. Specifically, iPSC-Mac from iPSC line#1 and 3 revealed CCL17 levels ranging from 200 to 260 pg/ml, while iPSC-Mac from line#2 demonstrated a higher secretion level of 1015 pg/ml (Fig. 5C).

Thus, all iPSC-Mac showed characteristic changes after exposure to pro- and anti-inflammatory stimuli and the adaptation of M1 or M2 phenotypes. However, the iPSC-Mac from the different lines displayed some variability in the levels of cytokine secretion.

### iPSC-Mac as a model system for the testing of immune-modulatory drugs

Ensuring the validity and reproducibility of model systems is crucial for testing the efficiency of novel immunotherapeutic strategies. As an alternative to artificial cell line models or primary cells, we evaluated the potential of our iPSC-Mac to show a dose-dependent response to LPS stimulation. More importantly, we examined the effect of the classical anti-inflammatory drug dexamethasone on the LPS-induced secretion of IL-6.

**Fig. 6:**
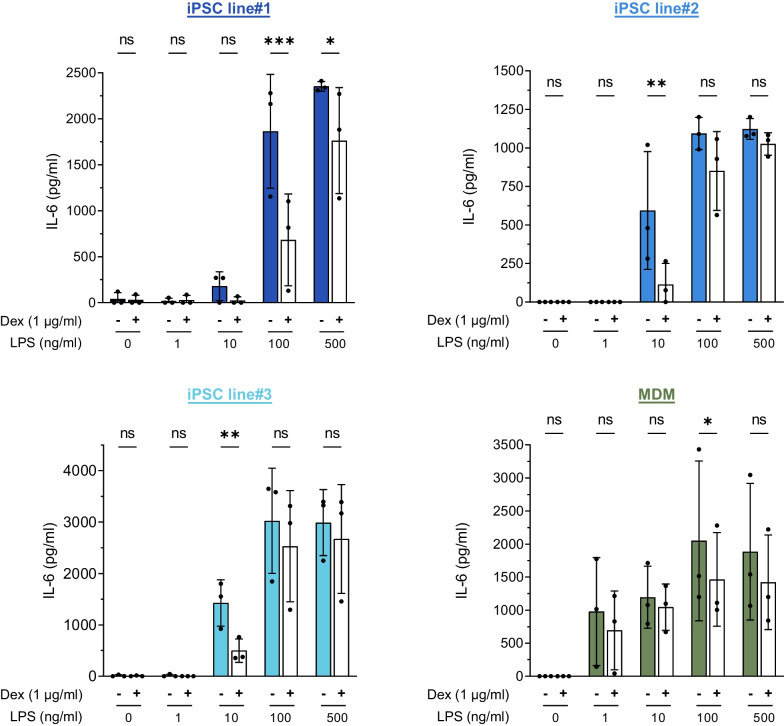
iPSC-Mac as a model system for the testing of immunodulatory drugs. Different iPSC-Mac as well as primary Mac were stimulated with increasing concentrations of LPS (0, 1, 10, 100 and 500 ng/ml) with or without the simultaneous addition of 1 ug/ml Dexamethasone for 4 h. Levels of IL-6 secretion were determined in supernatants using ELISA. (Individual values with mean ± SD, n = 3. Statistical Analysis was performed using two-way ANOVA with Sidak’s multi comparisons test (*p < 0.05, **p < 0.01, ***p < 0.001, ****p < 0.0001, ns denotes not significant)

All iPSC-Mac exhibited a dose-dependent response to increasing concentrations of LPS, as indicated by increasing secretion levels of IL-6 (Fig. 6). However, variations in the overall levels of secreted IL-6 were also observed. When stimulated with 500 ng/ml LPS, iPSC-Mac derived from line#2 showed lowest IL-6 levels with 1122.34 ± 67.54 pg/ml, iPSC-Mac from line#1 levels of 2352.83 ± 52.79 pg/ml and iPSC-Mac from line#3 the highest levels with 2989.56 ± 641.52 pg/ml. In comparison, primary macrophages displayed secretion levels of up to 1885.19 ± 1033.05 pg/ml. Irrespectively of these different levels of IL-6 secretion, all hiPSC lines showed a significant suppressive effect of dexamethasone on IL-6 secretion in a specific dose range of 10–100 ng/ml LPS stimulation (*p* = 0.0004 iPSC line#1 at 100 ng/ml LPS stimulation, *p* = 0.0032 iPSC line#2 at 10 ng/ml LPS stimulation and *p* = 0.0017 iPSC line#3 at 10 ng/ml LPS stimulation). Furthermore, we noted a significant effect of dexamethasone at 100 ng/ml LPS stimulation in MDMs, albeit with a larger *p* value of 0.0225 (Fig. 6).

In conclusion, these data illustrate that iPSC-Mac serve as a suitable model system for studying the effect of immune-modulatory drugs. Nevertheless, these findings highlight variations between the hiPSC lines in the optimal dose of stimulation.

## Discussion

To overcome the profound limitations associated with scalable, standardized, and off-the-shelf access to human macrophages, we here demonstrate an innovative protocol employing an easy to use, intermediate scale bioreactor platform, bridging the gap from the academic labs towards the clinical translation of macrophage and immune cell research. In fact, the introduced protocol is an advanced version of our previously published hiPSC differentiation scheme to the myeloid lineage [20, 23, 36], by introducing a serum- and feeder-free, suspension-based (3D) differentiation protocol in an intermediate-scale bioreactor platform applying 50 ml vessels. Employing the described semi-closed benchtop bioreactor system offers several advantages over the traditional 2D planar open culture system which has been utilized previously to drive macrophage production from hiPSCs [20, 46]. Of note, the benchtop bioreactors used in this study can provide regulated and consistent manufacturing schemes of the desired cell product with close monitoring of crucial manufacturing parameters (e.g. pH. temperature, CO2), while avoiding the high variability, and the human error introduced by open culture systems. While other studies have utilized the described benchtop bioreactor to scale up the production of other different somatic cell types, exploiting such platform for the production of blood related immune cells is lacking [5–7]. In that line, large stirred bioreactor tanks were previously employed to successfully scale up macrophage production from iPSCs to the needed clinical yield, however operating such platforms is cost intensive especially when it comes to either pre-clinical studies or process development approaches which are typically performed in academic research institutions [23, 36, 47]. Hence, the intermediate scale pipeline offers substantial advancements with respect to standardization compared to classical 2D-cell culture approaches, further facilitating macrophage translational research.

The organoid-based differentiation method reflects key stages of the early embryonic hematopoiesis indicated by the emergence of hemato-endothelial progenitor cells and their further hematopoietic commitment. Additionally, scRNA sequencing revealed that our generated iPSC-Mac have abundant expression of genes associated with yolk sac-like macrophages. This goes in line with other work that displayed the c-MYB independent heamtopoietic differentiation of iPSC to macrophages [41, 42]. Hence, such iPSC driven differentiations might give rise to valuable precursors under defined conditions, for the generation of tissue resident macrophages, such as microglia cells or alveolar macrophages [48, 49], collectively highlighting the numerous possible therapeutic and drug screening applications that can be facilitated by iPSC-Mac in the context of different tissue lineages.

However, a potential bottleneck that could hinder the application of macrophage is the limited bioavailability of human macrophages and the challenge of obtaining a relevant yield from such differentiation protocols in a cost-effective and timely manner. Therefore, by translating the small-scale suspension-based differentiation into the intermediate-scale bioreactor, we successfully improved the expected yield of macrophages production in a robust and rigorous manner from three different hiPSC lines, achieving an average weekly yield of 11.1–12.6 × 10^6^ iPSC-Mac /40 mL culture volume. Our production yield of 12 million cell/week was comparable to other studies employing scalable manufacturing methods. For instance, stirring cultures of the hemanoids in 120 ml industrial stirred bioreactor tanks resulted with a weekly harvest of approximately 1–4 × 10^7^ iPSC-Mac [23]. Using a different technique, scalable spinner cultures allowed for the production of 3 × 10^7^ monocyte per 6-well plate, that were further cultured in hM-CSF for 5–7 days to induce their differentiation towards mature macrophages [46]. Furthermore, Mathews et al., employed a combination of intermediate-scale bioreactor together with microcarriers to assist the expansion of hematopoietic organoids and scale the production of microglial cells. Their approach resulted with yields ranging between 5–40 × 10^6^ microglial cells per 1 million iPSC cells [48].

Of note and in contrast to most of the previously reported approaches, the intermediate scale differentiation approach highlighted here doesn’t necessitate the extra steps of macrophage progenitors’ separation, accumulation, expansion with external carriers or further differentiation to obtain the needed mature harvests of macrophages in meaningful yields and a timely manner. As in the described intermediate-scale approach, the clear advantage is the straightforward process, requiring essentially only the scalable platform and the culturing medium, without additional downstream culturing steps or supporting reagents to boost macrophage production. Thus, the stated protocol allows for mature, ready-to-use macrophage harvests within 10–15 days directly from the supernatants of the bioreactor tube, and the possibility to continuously harvest a defined cell product from the same differentiation process for up to 7 weeks.

Indeed, our freshly harvest of cells shed from the hemanoids showed a consistent typical mature macrophage-like phenotype and morphology comparable to that of the primary macrophages. The surface marker profile (level of expression) was even much more consistent compared to the variability of monocyte-derived macrophages derived from the different donors. This variability could be attributed to the donor variability, which is observed in the expression of CD14 and CD163. Furthermore, transcriptional analysis revealed that the harvested cells represent a high macrophage purity with minimal presence of contaminating cell types. Of note, the cells seemed to exhibit both pro-inflammatory nature with the observed expression of M1 genes in parallel to a prevailing expression of M2 genes. Also, the high expression of CD206 and low HLA-DR expression when compared to primary macrophages, supports a tendency towards a more anti-inflammatory phenotype. This was also seen in other studies [47] and can be explained (1) by the use of more M2 polarizing cytokine (hM-CSF) in the differentiation medium as well as (2) the tendency of the iPSC-Mac to resemble more primitive macrophages [50]. Of note, the protocol was rigorously reproduced across the three different hiPSC lines highlighting the strength, and the robustness of this method.

While the primary macrophages isolated from the different donors clearly demonstrated the expected donor-to-donor dependent variability in both phenotype and functionality, the generated iPSC-Mac were far more consistent in their performance with low variability across the different harvests and differentiations. Of note, iPSC-Mac also showed a higher functional capacity denoted by the higher ROS production, phagocytosis, and inflammatory cytokine secretion, which could be explained by the permittivity and the ontogenetic differences that iPSC-Mac harbor compared to adult primary macrophages (more primitive transcriptional fingerprint). This is in line with other reports, that demonstrated the faster and stronger reaction of iPSC-Mac to bacterial stimulation [35, 36], and their superior efferocytosis rate compared to MDM [47].

Our study also revealed that while iPSC-Mac can serve as a highly standardized cell type, nevertheless pinpointing the hiPSC line of choice is key for reproducibility. Interestingly, iPSC-Mac derived from different hiPSC lines/ donors revealed a line-dependent variability in the different functional assessments. Additionally, this was further confirmed with scRNA seq data that displayed different clustering patterns of the iPSC-Mac derived from the separate lines/donors. While clearly all iPSC-Mac populations demonstrate a typical macrophage core transcriptional program, the ratio between clusters associated with pro- or anti-inflammatory activation stages showed some variability between the different lines. Here, iPSC line #1 and 3 demonstrated a higher abundance of cells associated with more pro-inflammatory clusters, whereas iPSC-Mac derived from iPSC line#2 showed a higher prevalence of cells associated with anti-inflammatory genes. This observation goes in hand with the fact, that iPSC-Mac from line#2 also displayed a higher level of CCL17 secretion observed after IL-4 stimulation. In summary, we observed minor differences in the pro- and anti-inflammatory macrophage clusters between the different iPSC lines. To unveil the significance of these differences further follow-up studies are needed to better interpret potential discrepancies and how they relate to their primary counterparts.

The stated line-dependent variability might suggest that hiPSC reprograming is maintaining a certain epigenetic/genetic landscape of the donor somatic cells, which necessitates further work to deepen our knowledge on the full impact of reprogramming processes on the maintenance or alteration of the original donor’s fingerprint. Additionally, such variation also highlights the potential need to use a defined portfolio of iPSC-lines when applying iPSC derivatives for different therapeutic and non-therapeutic applications to better reflect the population diversity.

Independent of the differences in the transcriptional activation state of the freshly harvested iPSC-Mac from the different lines, it was crucial to demonstrate that our platform can give rise to macrophages capable of recapitulating macrophage plasticity, given the well-known roles of macrophage polarization stages in human health and disease [51]. Indeed, when the physiological cues were mimicked, iPSC-Mac from the three lines were all able to adopt towards both, pro- and anti-inflammatory states with characteristic phenotypic changes, that overlap with previous studies highlighting the plasticity of iPSC-Mac [52, 53].

Another growing application for human macrophages is their use in high-throughput drug screening purposes, here again such scalable production pipelines would be of great value. To further elaborate on that, we have established an in-vitro setup that can screen for the activity of different immunomodulatory agents.

While other studies have either exploited this bench top suspension culturing platform for iPSC expansion, or to scale-up iPSC differentiation towards the hepatic, neuronal or cardiac lineages [29, 30, 33], we here provide an additional translational application by tailoring a robust hematopoietic differentiation protocol for continuous macrophage production. Of note, previous work could also reveal the continuous generation of other myeloid lineages using a similar, yet small scale differentiation pipelines. For instance, different combinations of instructing cytokines were used for the generation of granulocytes [20], erythrocytes [54], progenitors [55] and other blood immune cells (not yet published). Given the similarities in the origin and design of the intermediate scale differentiation protocol with the previously published work we assume, that hematopoietic differentiation in the intermediate scale platform would be also possible for the aforementioned cell lineages [20, 54, 55]. Hence, this technology can fulfill the need of recruiting large numbers of defined and functional immune cells in a reproducible and affordable manner, with a robust easy to use protocol, paving the way to translate macrophage and immune cell research in different therapeutic, drug screening applications, and beyond.

## Conclusion

Our study addresses the gap for intermediate-scale production of hematopoietic organoids, and standardized production of macrophages in bench top bioreactors. This introduced novel method exploited the use of an intermediate-scale bioreactor platform to generate standardized macrophages in a robust and reproducible manner. Employing this technique provides ready to use macrophages that are functionally active and require no downstream maturation steps, making them highly desirable for both therapeutic and non-therapeutic applications.

## Supplementary Information

Additional file 1: Fig. S1. Organoid-based production of iPSC-Mac in intermediate scale bioreactors recapitulates embryonic hematopoietic development. **A** Analysis of KDR/VEGFR and CD45/PPTRC at different stages of differentiation as well as in iPSC-derived macrophages (iPSC-Mac) by qRT-PCR. Values are represented as relative RNA expression to GAPDH (housekeeping gene) (individual values with mean ± SD, iPSC line#1: blue square, and iPSC line#2: purple dots, n = 2–3 per line, n.d. indicates detection limit of the target gene) **B** and **C** Flow cytometric analysis of CD34, CD144, CD43 and CD45 expression during early hematopoietic differentiation. Hemanoids were dissociated and analyzed on day 4, 7 and 10 of mesoderm priming as well as during hematopoietic differentiation after they initiated production of iPSC-derived Mac. **B** Representative FACs plots CD34^+^/CD144^+^/CD45^−^ Hemato-endothelial progenitors, CD34^+^/CD144-/CD43^+^ early hematopoietic progenitors and CD34^+^/CD144-/CD45^+^ hematopoetic progenitors during mesoderm priming and early hematopoietic differentiation (representative data shown for iPSC line#1). **C** Representative gating strategy: Populations were pre-gated for viable cells (FSC/SSC), single cells (FSC-A/FSC-H), CD34^+^ cells (CD34-FITC/autofluorescence), viability staining (Zombie-Aqua/PB450-A). **D** Immunohistochemical analysis of VE-Cadherin/CD144, VEGFR2 and CD45 expression in hemanoids derived from day 4, 7 and 10 of mesoderm priming as well as from hemanoids during hematopoietic differentiation after they initiated production of iPSC-derived Mac (day 7–10). Arrows indicate characteristic regions (scale bar = 200 µm, data shown for iPSC line#2, representative of n = 2).

Additional file 2: Fig. S2. Phenotypic characterization of iPSC-derived macrophages continuously produced in intermediate scale bioreactors (I). **A** Representative brightfield images and cytospin staining for iPSC-derived macrophages (iPSC-Mac) derived from harvest 1b, 3 and 5 for iPSC line#2 (left) and iPSC line#3 (right), respectively. **B** Representative gating strategy for flow cytometric analysis of iPSC-Mac. Populations were pre-gated for viable cells (FSC7SSC) and single cells (SSC-A/SSC-H). **C** Frequencies of CD11b, CD14, CD163, CD86, CD206, and HLA-DR expression on iPSC-Mac from all three iPSC lines and all different harvests (Individual values with mean).

Additional file 3: Fig. S3. Phenotypic characterization of iPSC-derived macrophages continuously produced in intermediate scale bioreactors (II). **A** Representative brightfield images and cytospin staining for iPSC-derived macrophages (iPSC-Mac) derived from the three different lines. **B** Gating strategy and flow cytometric analysis of CD11b, CD14, CD163, CD86, HLA-DR and CD206 expression on primary monocyte-derived macrophages (MDM, representative data of n = 5). **C** Frequency of CD86, CD206 and HLA-DR positive cells derived from different harvests/differentiations of the three different hiPSC lines as well as primary monocyte-derived macrophages. Individual values with mean ± SD, n = 5–15). Coefficient of variation (CV) is given for all values.

Additional file 4: Fig. S4. Heat map of yolk sac macrophages genes. A heat map illustrating a list of genes associated with primitive yolk-sac macrophages (adapted from [36]).

Additional file 5: Fig. S5. iPSC-Mac demonstrate important pro-inflammatory functionality. **A** Phagocytosis of pHrodo™ Red E. coli BioParticles. Frequency of pHrodo Red^+^ cells derived from different harvests/differentiations of the three different hiPSC lines (Individual values with mean). **B** Production of reactive oxygen species (ROS) by macrophages from the different sources. Fold change of Rhodamine mean fluorescence intensity (MFI) for iPSC-Mac derived from different harvests/differentiations of the three different hiPSC lines (Individual values with mean, dotted line indicates “1”).

Additional file 6: Fig. S6. Polarization of iPSC-Mac into different pro- and anti-inflammatory activation stages. Changes in surface marker expression of CD64, HLA-DR, CD86 and CD206 24 h after polarization analyzed by flow cytometry (Representative data of n = 3–4 shown for all three iPSC lines).

Additional file 7.

Additional file 8.

Additional file 9.

## Data Availability

All the presented data are available for consultation. scRNA sequencing datasets generated as part of this study are available at the NCBI GEO repository (NCBI Accession: GSE268458). All stable reagents utilized/generated in this study could be provided upon request by the lead contact person, upon the completion of the necessary material transfer agreement. For further information and requests for the indicated reagents, all requests should be directed and will be fulfilled by Prof. Dr. Nico Lachmann (Lachmann.Nico@mh-hannover.de).

## Abbreviations

iPSC: Induced pluripotent stem cell
iPSC-Mac: iPSC-derived macrophages
