# Supplementary figures and images for: Standardized generation of human iPSC-derived hematopoietic organoids and macrophages utilizing a benchtop bioreactor platform under fully defined conditions
Source: Stem Cell Res Ther. 2024 Jun 18;15:171. doi: 10.1186/s13287-024-03785-2 (PMC11184717; doi:10.1186/s13287-024-03785-2)

Supplement Figure 1:

A

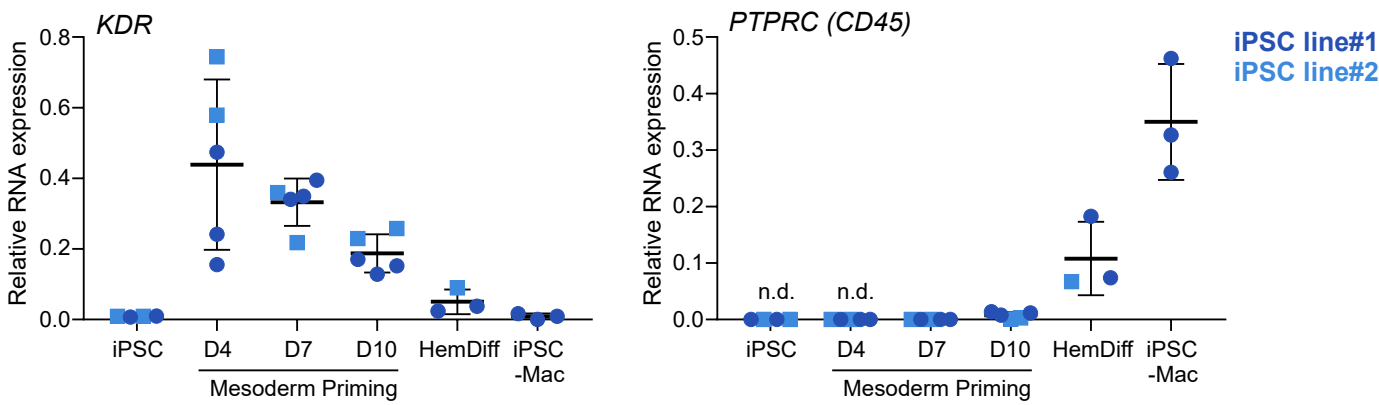

B

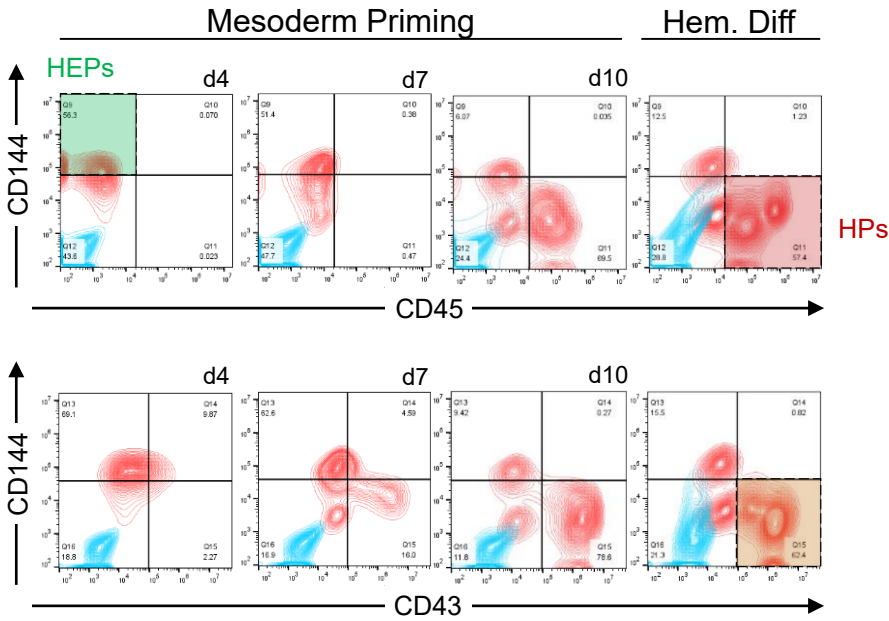

C

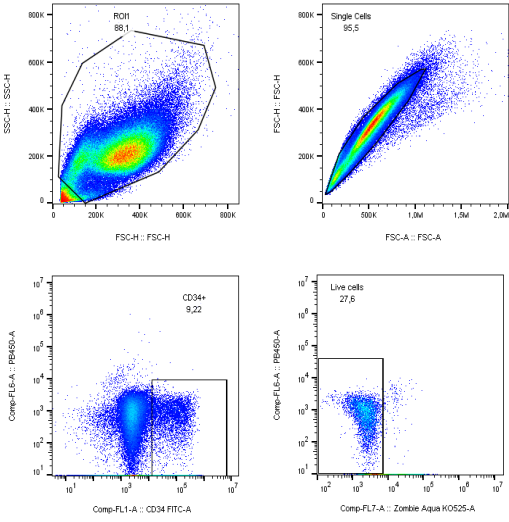

D

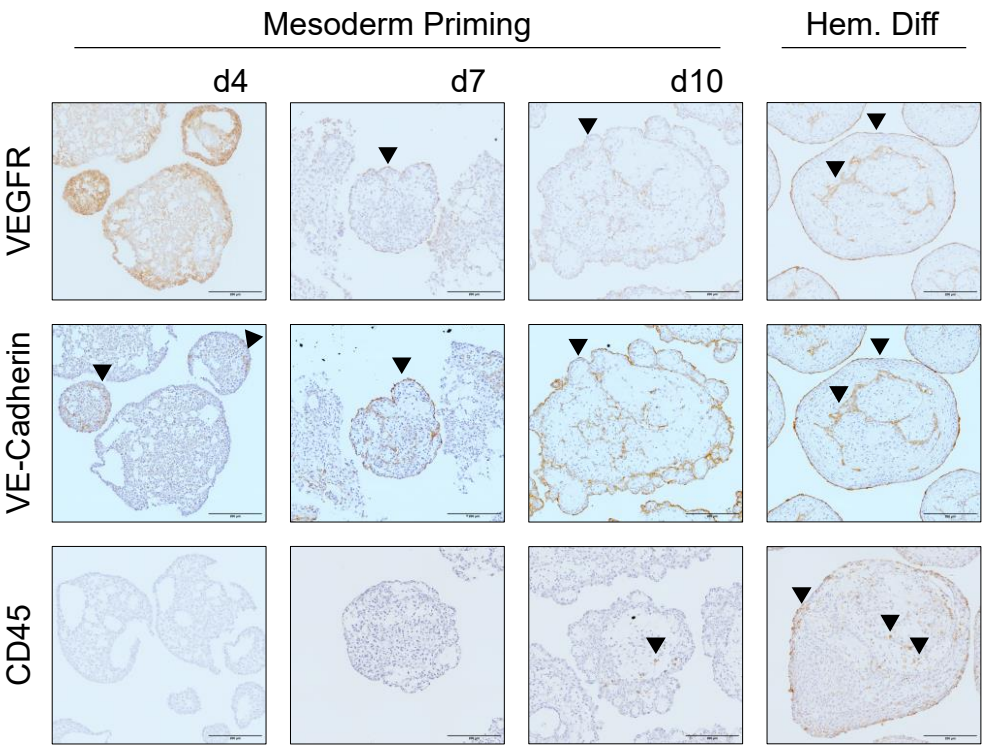

Supplement: Supplementary file 1 — Additional file 1: Fig. S1. Organoid-based production of iPSC-Mac in intermediate scale bioreactors recapitulates embryonic hematopoietic development. A Analysis of KDR/VEGFR and CD45/PPTRC at different stages of differentiation as well as in iPSC-derived macrophages (iPSC-Mac) by qRT-PCR. Values are represented as relative RNA expression to GAPDH (housekeeping gene) (individual values with mean ± SD, iPSC line#1: blue square, and iPSC line#2: purple dots, n = 2–3 per line, n.d. indicates detection limit of the target gene) B and C Flow cytometric analysis of CD34, CD144, CD43 and CD45 expression during early hematopoietic differentiation. Hemanoids were dissociated and analyzed on day 4, 7 and 10 of mesoderm priming as well as during hematopoietic differentiation after they initiated production of iPSC-derived Mac. B Representative FACs plots CD34+/CD144+/CD45− Hemato-endothelial progenitors, CD34+/CD144-/CD43+ early hematopoietic progenitors and CD34+/CD144-/CD45+ hematopoetic progenitors during mesoderm priming and early hematopoietic differentiation (representative data shown for iPSC line#1). C Representative gating strategy: Populations were pre-gated for viable cells (FSC/SSC), single cells (FSC-A/FSC-H), CD34+ cells (CD34-FITC/autofluorescence), viability staining (Zombie-Aqua/PB450-A). D Immunohistochemical analysis of VE-Cadherin/CD144, VEGFR2 and CD45 expression in hemanoids derived from day 4, 7 and 10 of mesoderm priming as well as from hemanoids during hematopoietic differentiation after they initiated production of iPSC-derived Mac (day 7–10). Arrows indicate characteristic regions (scale bar = 200 µm, data shown for iPSC line#2, representative of n = 2). [file 13287_2024_3785_MOESM1_ESM.pdf]

Supplement Figure 2:

A

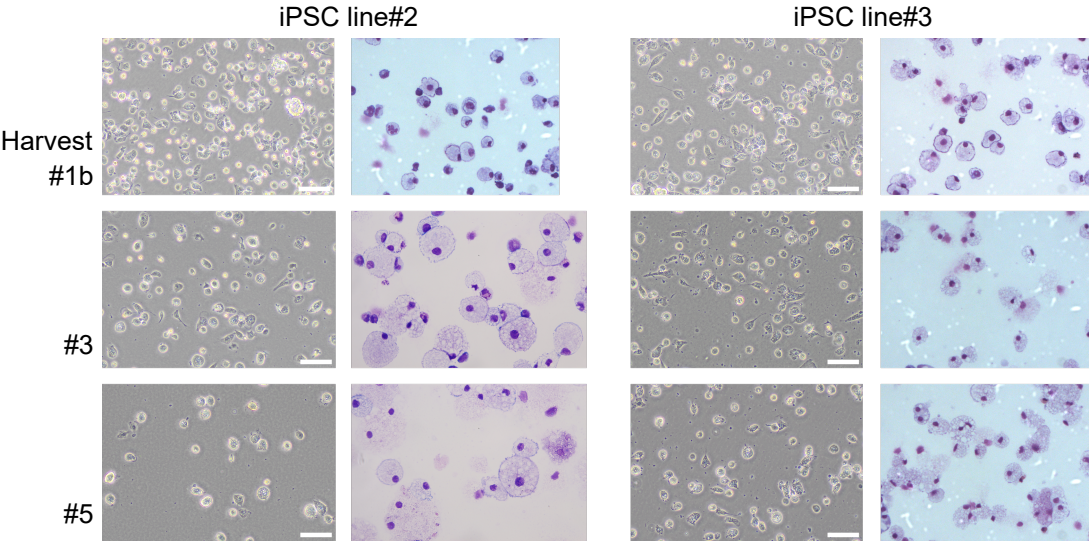

B

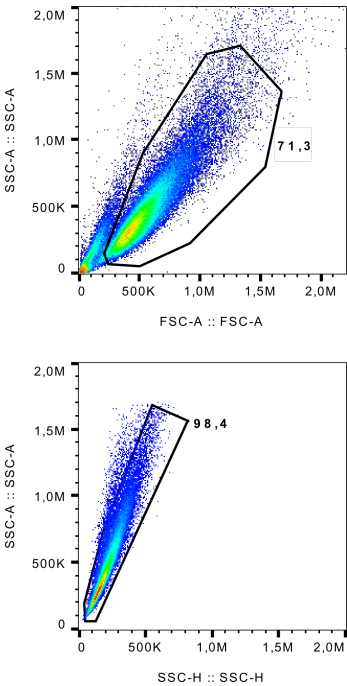

C

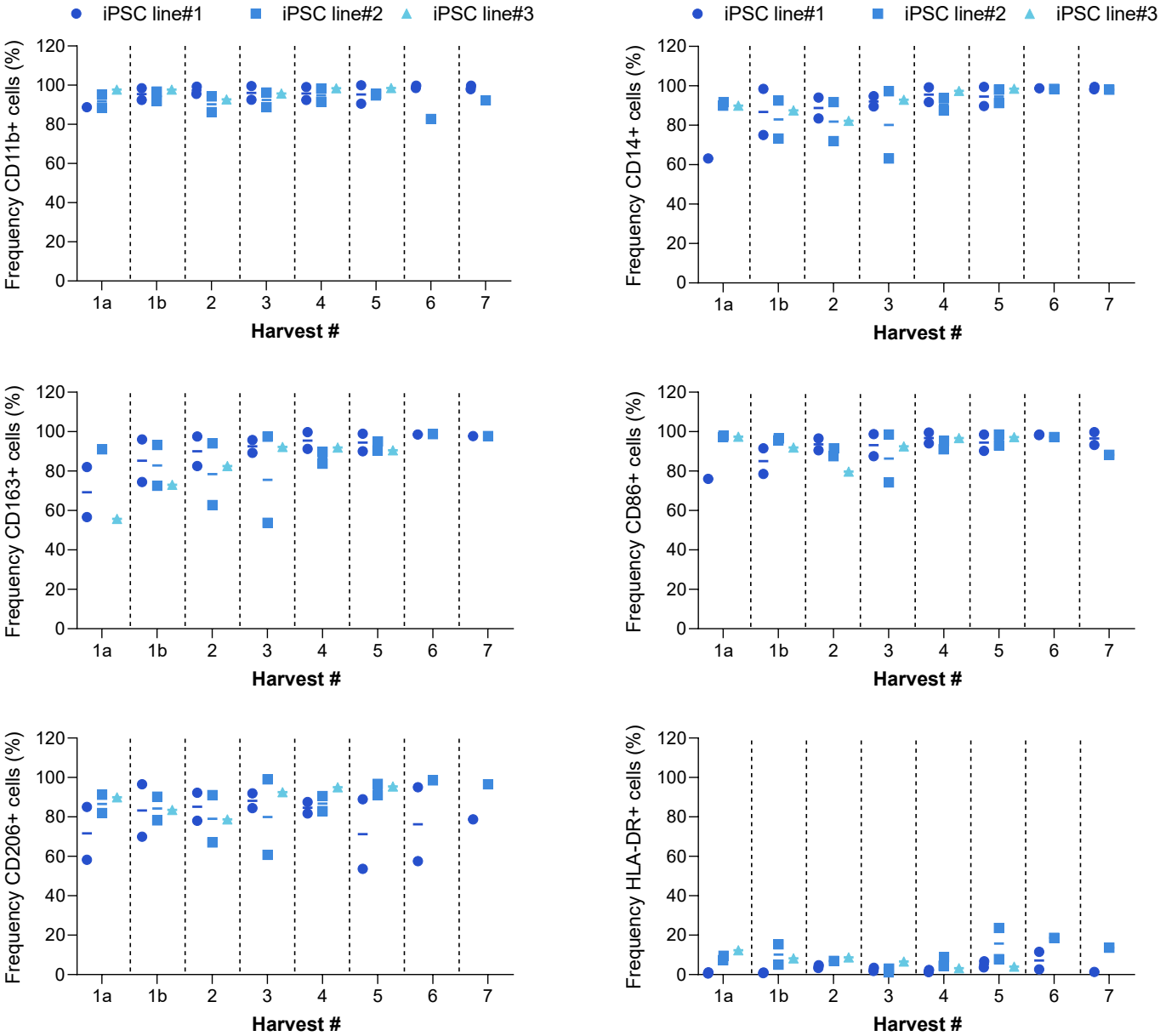

Supplement: Supplementary file 2 — Additional file 2: Fig. S2. Phenotypic characterization of iPSC-derived macrophages continuously produced in intermediate scale bioreactors (I). A Representative brightfield images and cytospin staining for iPSC-derived macrophages (iPSC-Mac) derived from harvest 1b, 3 and 5 for iPSC line#2 (left) and iPSC line#3 (right), respectively. B Representative gating strategy for flow cytometric analysis of iPSC-Mac. Populations were pre-gated for viable cells (FSC7SSC) and single cells (SSC-A/SSC-H). C Frequencies of CD11b, CD14, CD163, CD86, CD206, and HLA-DR expression on iPSC-Mac from all three iPSC lines and all different harvests (Individual values with mean). [file 13287_2024_3785_MOESM2_ESM.pdf]

Supplement Figure 3:

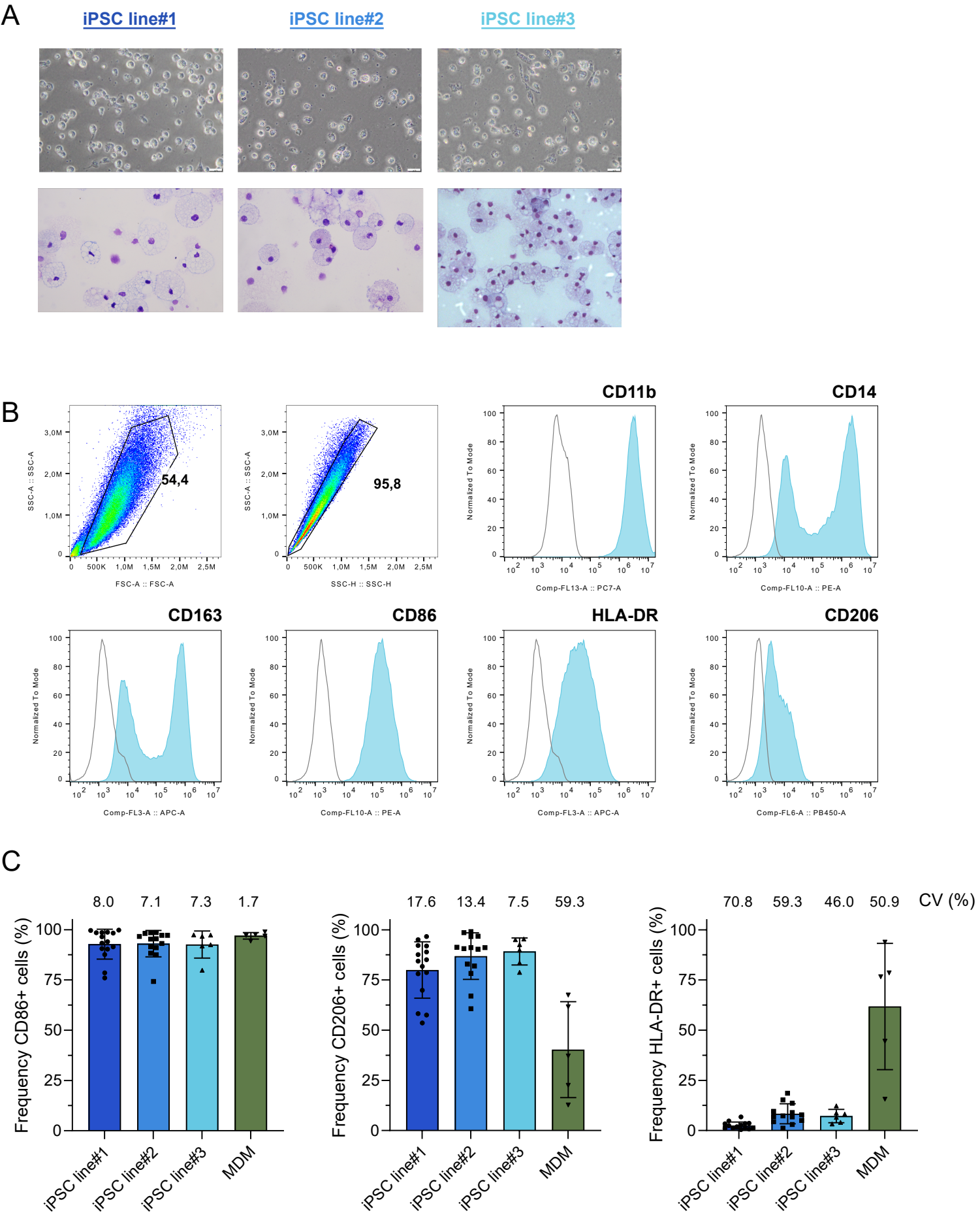

Supplement: Supplementary file 3 — Additional file 3: Fig. S3. Phenotypic characterization of iPSC-derived macrophages continuously produced in intermediate scale bioreactors (II). A Representative brightfield images and cytospin staining for iPSC-derived macrophages (iPSC-Mac) derived from the three different lines. B Gating strategy and flow cytometric analysis of CD11b, CD14, CD163, CD86, HLA-DR and CD206 expression on primary monocyte-derived macrophages (MDM, representative data of n = 5). C Frequency of CD86, CD206 and HLA-DR positive cells derived from different harvests/differentiations of the three different hiPSC lines as well as primary monocyte-derived macrophages. Individual values with mean ± SD, n = 5–15). Coefficient of variation (CV) is given for all values. [file 13287_2024_3785_MOESM3_ESM.pdf]

Supplement Figure 4:

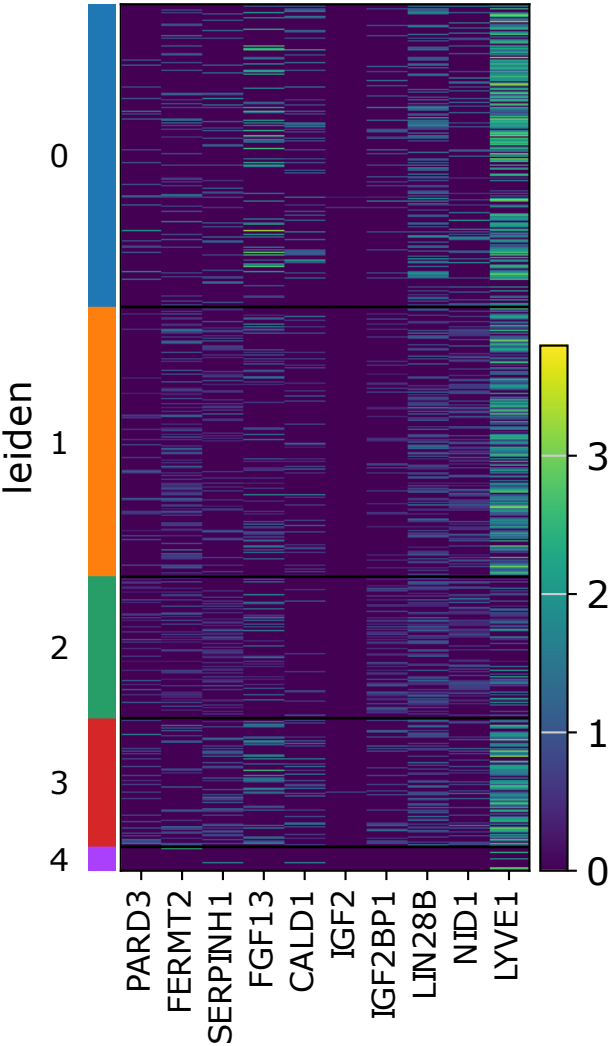

Supplement: Supplementary file 4 — Additional file 4: Fig. S4. Heat map of yolk sac macrophages genes. A heat map illustrating a list of genes associated with primitive yolk-sac macrophages (adapted from [36]). [file 13287_2024_3785_MOESM4_ESM.pdf]

Supplement Figure 5:

A

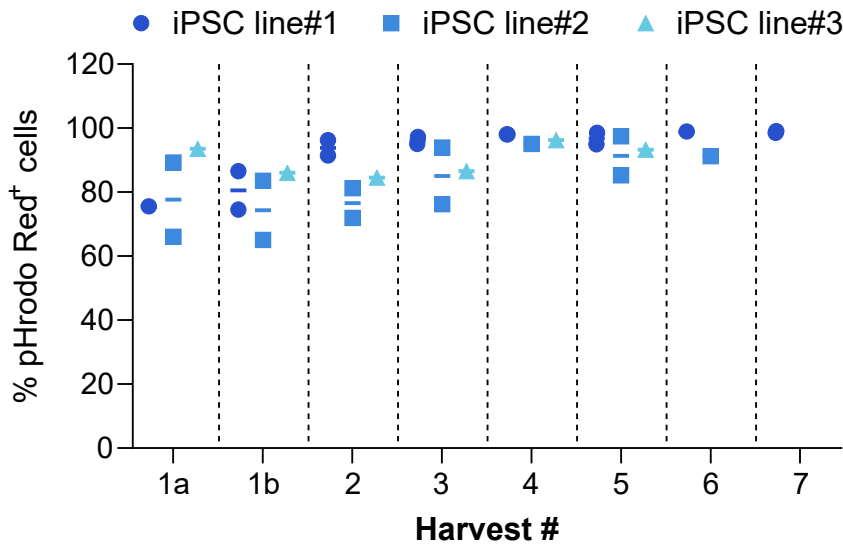

B

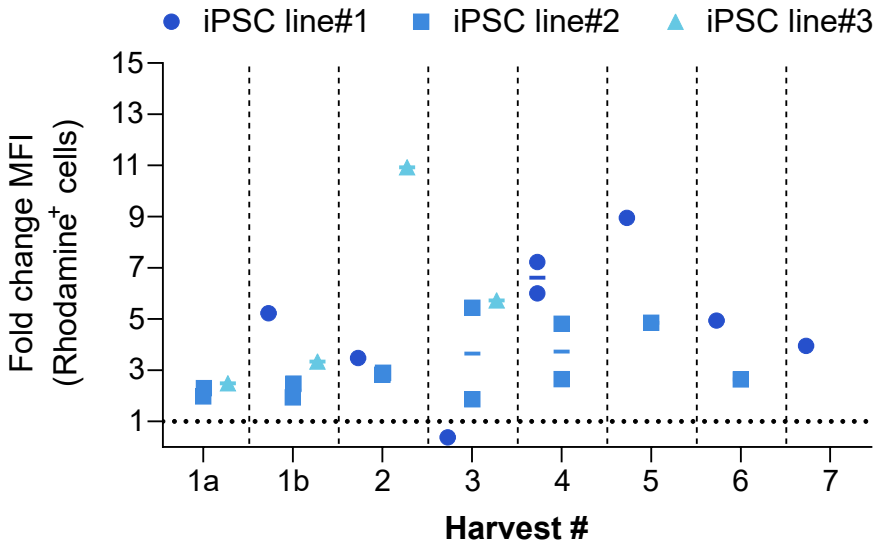

Supplement: Supplementary file 5 — Additional file 5: Fig. S5. iPSC-Mac demonstrate important pro-inflammatory functionality. A Phagocytosis of pHrodo™ Red E. coli BioParticles. Frequency of pHrodo Red+ cells derived from different harvests/differentiations of the three different hiPSC lines (Individual values with mean). B Production of reactive oxygen species (ROS) by macrophages from the different sources. Fold change of Rhodamine mean fluorescence intensity (MFI) for iPSC-Mac derived from different harvests/differentiations of the three different hiPSC lines (Individual values with mean, dotted line indicates “1”). [file 13287_2024_3785_MOESM5_ESM.pdf]

Supplement Figure 6:

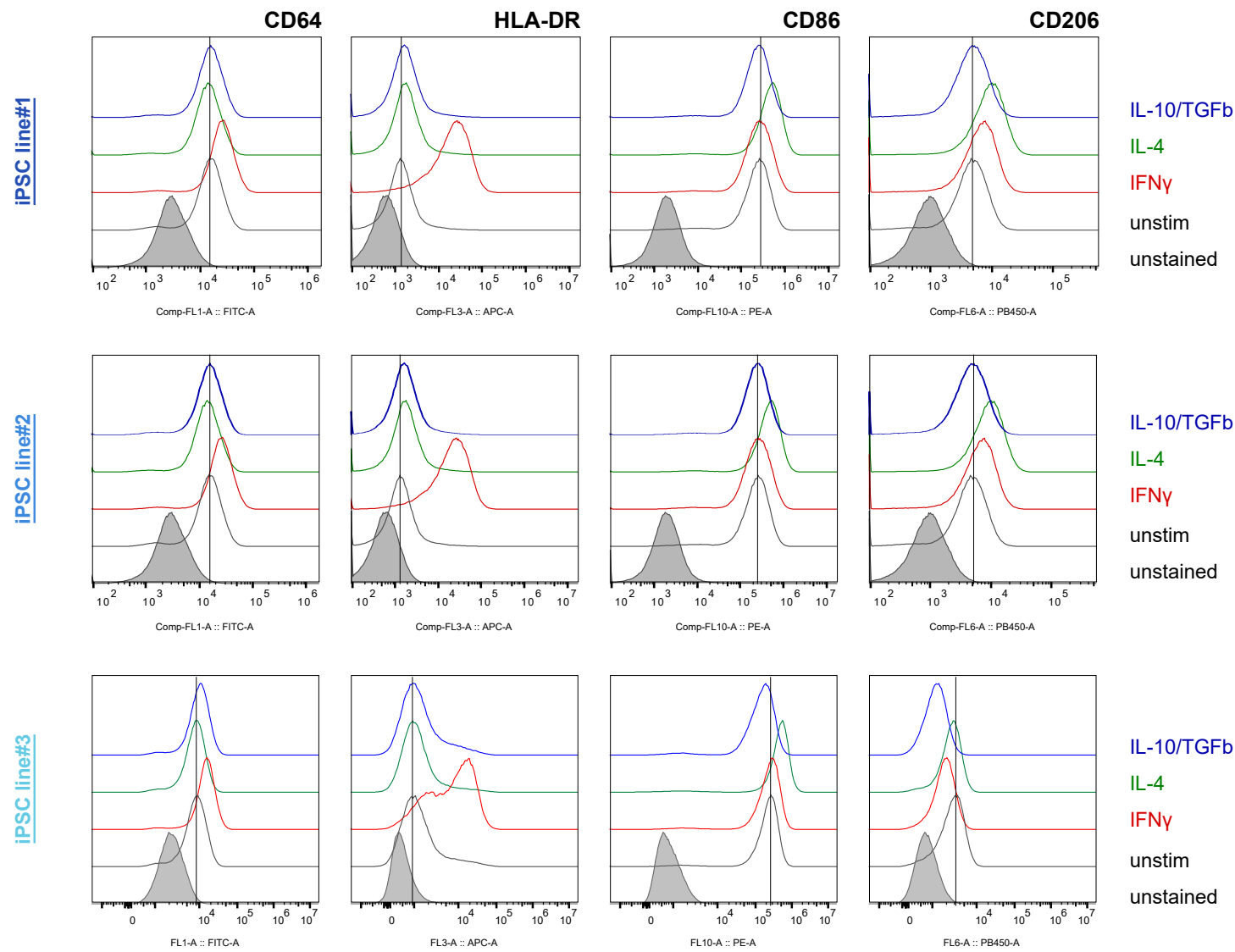

Supplement: Supplementary file 6 — Additional file 6: Fig. S6. Polarization of iPSC-Mac into different pro- and anti-inflammatory activation stages. Changes in surface marker expression of CD64, HLA-DR, CD86 and CD206 24 h after polarization analyzed by flow cytometry (Representative data of n = 3–4 shown for all three iPSC lines). [file 13287_2024_3785_MOESM6_ESM.pdf]
