# Supplementary material for: Standardized generation of human iPSC-derived hematopoietic organoids and macrophages utilizing a benchtop bioreactor platform under fully defined conditions
Source: Stem Cell Res Ther. 2024 Jun 18;15:171. doi: 10.1186/s13287-024-03785-2 (PMC11184717; doi:10.1186/s13287-024-03785-2)
Supplement: Supplementary file 7 — Additional file 7. [file 13287_2024_3785_MOESM7_ESM.html]

None 
 
  
 

 CelltypeIdentifcation_202401_lessClusters  


 


 

     

 

 
     
     
     
      

  

 

 

 

 

 
 
 
 
 
 
 
 In [1]: 
 
      
    %%bash
 echo     "Current Conda environment:   ${  CONDA_DEFAULT_ENV  }  " 
  

      
 
 
 

 
 
 


 
 
    
      


 
 Current Conda environment: scRNA2023
 
 
 

 

 

  
 
 
 
 
 In [1]: 
 
      
     %  matplotlib  inline
 #%load_ext memory_profiler 
 import   sys 
 sys  .  path  .  append  (  "/mnt/dzl_bioinf/exec/miniconda/miniconda3/envs/scRNA/lib/python3.7/site-packages'"  ) 
 import   os 
 import   numpy   as   np 
 import   pandas   as   pd 
 import   scanpy   as   sc 
 import   datetime 
 import   scipy 
 import   seaborn   as   sb 
 import   matplotlib.pyplot   as   plt 
 from   matplotlib   import   colors 

 os  .  chdir  (  "/mnt/dzl_bioinf/gaedckes/scRNASeq/Macrophagen_AGLachmann_202311/"  ) 

 sc  .  settings  .  verbosity   =   0           # verbosity: errors (0), warnings (1), info (2), hints (3) 
 sc  .  logging  .  print_versions  () 
 sc  .  set_figure_params  (  scanpy  =  True  ,   dpi  =  100  ,   dpi_save  =  150  , 
                          frameon  =  True  ,   vector_friendly  =  True  ,  
                          fontsize  =  14  ,   color_map  =  None  ,   format  =  'pdf'  , 
                          transparent  =  True   ) 
 sc  .  _settings  .  n_jobs   =   5 
 sc  .  _settings  .  max_memory   =   80   #GB 
 plt  .  rcParams  [  'figure.figsize'  ]  =  (  5  ,  5  )   #rescale figures 
 sc  .  _settings  .  savefigs   =   True 
 sc  .  settings  .  autosave   =   True 
 sc  .  _settings  .  figdir   =   "./figures_lessClusters/" 
  

      
 
 
 

 
 
 


 
 
    
      


 
 -----
anndata     0.7.8
scanpy      1.9.3
-----
PIL                         9.4.0
asciitree                   NA
asttokens                   NA
backcall                    0.2.0
beta_ufunc                  NA
binom_ufunc                 NA
bottleneck                  1.3.5
cffi                        1.15.1
cloudpickle                 2.2.1
colorama                    0.4.6
comm                        0.1.2
cycler                      0.10.0
cython_runtime              NA
cytoolz                     0.12.0
dask                        2023.4.1
dateutil                    2.8.2
debugpy                     1.5.1
decorator                   5.1.1
defusedxml                  0.7.1
entrypoints                 0.4
executing                   0.8.3
fasteners                   NA
h5py                        3.1.0
hypergeom_ufunc             NA
igraph                      0.9.10
importlib_resources         NA
ipykernel                   6.19.2
ipython_genutils            0.2.0
ipywidgets                  8.0.4
jedi                        0.18.1
jinja2                      3.1.2
joblib                      1.2.0
kiwisolver                  1.4.4
leidenalg                   0.8.10
llvmlite                    0.40.0
markupsafe                  2.1.1
matplotlib                  3.7.1
matplotlib_inline           0.1.6
mpl_toolkits                NA
mpmath                      1.3.0
msgpack                     1.0.3
natsort                     7.1.1
nbinom_ufunc                NA
ncf_ufunc                   NA
numba                       0.57.1
numcodecs                   0.11.0
numexpr                     2.8.4
numpy                       1.24.4
nvfuser                     NA
opt_einsum                  v3.3.0
packaging                   23.0
pandas                      1.5.3
parso                       0.8.3
pexpect                     4.8.0
pickleshare                 0.7.5
pkg_resources               NA
platformdirs                2.5.2
prompt_toolkit              3.0.36
psutil                      5.9.0
ptyprocess                  0.7.0
pure_eval                   0.2.2
pydev_ipython               NA
pydevconsole                NA
pydevd                      2.6.0
pydevd_concurrency_analyser NA
pydevd_file_utils           NA
pydevd_plugins              NA
pydevd_tracing              NA
pygments                    2.15.1
pyparsing                   3.0.9
pytz                        2022.7
scipy                       1.9.3
seaborn                     0.12.2
session_info                1.0.0
setuptools                  67.8.0
six                         1.16.0
sklearn                     1.1.3
sparse                      0.14.0
sphinxcontrib               NA
stack_data                  0.2.0
statsmodels                 0.13.5
sympy                       1.12
tblib                       1.7.0
texttable                   1.6.4
threadpoolctl               2.2.0
tlz                         0.12.0
toolz                       0.12.0
torch                       2.0.1+cu117
tornado                     6.2
tqdm                        4.66.1
traitlets                   5.7.1
typing_extensions           NA
wcwidth                     0.2.5
yaml                        6.0
zarr                        2.13.3
zipp                        NA
zmq                         25.0.2
zoneinfo                    NA
-----
IPython             8.12.0
jupyter_client      8.1.0
jupyter_core        5.3.0
notebook            6.5.4
-----
Python 3.9.16 (main, Mar  8 2023, 14:00:05) [GCC 11.2.0]
Linux-5.4.0-122-generic-x86_64-with-glibc2.31
-----
Session information updated at 2024-02-16 11:14
 
 
 

 

 

  
 
 
 
 
 In [3]: 
 
      
     adata   =   sc  .  read  (  "/mnt/dzl_bioinf/gaedckes/scRNASeq/Macrophagen_AGLachmann_202311/results/scAnalyse_preprocessed_20231204.h5ad"  ) 
  

      
 
 
 

 
 
 
 
 
  
  
 UMAP ¶  
 
 
 
  
 
 
 
 
 In [4]: 
 
      
     adata 
  

      
 
 
 

 
 
 


 
 
    
     Out[4]: 


 
 AnnData object with n_obs × n_vars = 10396 × 29143
    obs: 'sample', 'batch', 'group', 'n_genes_by_counts', 'total_counts', 'total_counts_mito', 'pct_counts_mito', 'n_counts', 'predicted_doublets', 'doublet_scores', 'size_factors', 'leiden'
    var: 'gene_ids', 'feature_types', 'mito', 'n_cells_by_counts', 'mean_counts', 'pct_dropout_by_counts', 'total_counts', 'n_counts', 'highly_variable', 'means', 'dispersions', 'dispersions_norm'
    uns: 'batch_colors', 'group_colors', 'hvg', 'leiden', 'leiden_colors', 'log1p', 'neighbors', 'neighbors_harmony', 'predicted_doublets_colors', 'sample_colors', 'umap'
    obsm: 'X_pca', 'X_pca_harmony', 'X_umap'
    layers: 'counts'
    obsp: 'connectivities', 'distances', 'neighbors_harmony_connectivities', 'neighbors_harmony_distances' 
 

 

 

 

  
 
 
 
 
 In [5]: 
 
      
     sc  .  tl  .  leiden  (  adata  ,   resolution  =  0.2  ) 
 sc  .  pl  .  umap  (  adata  ,   color  =  "leiden"  ,   save  =  "_clusters"  ) 
  

      
 
 
 

 
 
 


 
 
    
      


 
 /mnt/dzl_bioinf/exec/miniconda/miniconda3/envs/scRNA2023/lib/python3.9/site-packages/scanpy/plotting/_tools/scatterplots.py:392: UserWarning: No data for colormapping provided via 'c'. Parameters 'cmap' will be ignored
  cax = scatter(
 
 
 
 
    
      


 
 
 

 

 

 

  
 
 
 
 
 In [6]: 
 
      
     #clustering is done in preprocesing! 
 #sc.tl.leiden(adata, resolution=1)  
 print  (  "number of cells per Leiden-Cluster"  ) 
 adata  .  obs  [  'leiden'  ]  .  value_counts  () 
  

      
 
 
 

 
 
 


 
 
    
      


 
 number of cells per Leiden-Cluster
 
 
 
 
    
     Out[6]: 


 
 0    3632
1    3236
2    1698
3    1544
4     286
Name: leiden, dtype: int64 
 

 

 

 

  
 
 
 
 
 In [7]: 
 
      
     sc  .  pl  .  umap  (  adata  ,   color  =  [  "leiden"  ],  wspace  =  0.3  ,   legend_loc   =  'on data'  ) 
 sc  .  pl  .  umap  (  adata  ,   color  =  [  "leiden"  ,   "group"  ],  wspace  =  0.3  ,    save  =  "_clusters_group"  ) 
  

      
 
 
 

 
 
 


 
 
    
      


 
 /mnt/dzl_bioinf/exec/miniconda/miniconda3/envs/scRNA2023/lib/python3.9/site-packages/scanpy/plotting/_tools/scatterplots.py:392: UserWarning: No data for colormapping provided via 'c'. Parameters 'cmap' will be ignored
  cax = scatter(
 
 
 
 
    
      


 
 
 

 
 
    
      


 
 /mnt/dzl_bioinf/exec/miniconda/miniconda3/envs/scRNA2023/lib/python3.9/site-packages/scanpy/plotting/_tools/scatterplots.py:392: UserWarning: No data for colormapping provided via 'c'. Parameters 'cmap' will be ignored
  cax = scatter(
/mnt/dzl_bioinf/exec/miniconda/miniconda3/envs/scRNA2023/lib/python3.9/site-packages/scanpy/plotting/_tools/scatterplots.py:392: UserWarning: No data for colormapping provided via 'c'. Parameters 'cmap' will be ignored
  cax = scatter(
 
 
 
 
    
      


 
 
 

 

 

 

  
 
 
 
 
 In [8]: 
 
      
     sc  .  pl  .  umap  (  adata  [  adata  .  obs  .  group   ==   "NIH"  ],   color  =  [  "leiden"  ,   "group"  ],  wspace  =  0.3  ,   save  =  "_group_NIH"  ) 
 sc  .  pl  .  umap  (  adata  [  adata  .  obs  .  group   ==   "Pheonix"  ],   color  =  [  "leiden"  ,   "group"  ],  wspace  =  0.3  ,   save  =  "_group_Pheonix"  ) 
 sc  .  pl  .  umap  (  adata  [  adata  .  obs  .  group   ==   "hCD34iPSC11"  ],   color  =  [  "leiden"  ,   "group"  ],  wspace  =  0.3  ,   save  =  "_group_hCDf"  ) 
  

      
 
 
 

 
 
 


 
 
    
      


 
 /mnt/dzl_bioinf/exec/miniconda/miniconda3/envs/scRNA2023/lib/python3.9/site-packages/scanpy/plotting/_utils.py:430: ImplicitModificationWarning: Trying to modify attribute `._uns` of view, initializing view as actual.
  adata.uns[value_to_plot + '_colors'] = colors_list
/mnt/dzl_bioinf/exec/miniconda/miniconda3/envs/scRNA2023/lib/python3.9/site-packages/scanpy/plotting/_tools/scatterplots.py:392: UserWarning: No data for colormapping provided via 'c'. Parameters 'cmap' will be ignored
  cax = scatter(
/mnt/dzl_bioinf/exec/miniconda/miniconda3/envs/scRNA2023/lib/python3.9/site-packages/scanpy/plotting/_tools/scatterplots.py:392: UserWarning: No data for colormapping provided via 'c'. Parameters 'cmap' will be ignored
  cax = scatter(
 
 
 
 
    
      


 
 
 

 
 
    
      


 
 /mnt/dzl_bioinf/exec/miniconda/miniconda3/envs/scRNA2023/lib/python3.9/site-packages/scanpy/plotting/_utils.py:430: ImplicitModificationWarning: Trying to modify attribute `._uns` of view, initializing view as actual.
  adata.uns[value_to_plot + '_colors'] = colors_list
/mnt/dzl_bioinf/exec/miniconda/miniconda3/envs/scRNA2023/lib/python3.9/site-packages/scanpy/plotting/_tools/scatterplots.py:392: UserWarning: No data for colormapping provided via 'c'. Parameters 'cmap' will be ignored
  cax = scatter(
/mnt/dzl_bioinf/exec/miniconda/miniconda3/envs/scRNA2023/lib/python3.9/site-packages/scanpy/plotting/_tools/scatterplots.py:392: UserWarning: No data for colormapping provided via 'c'. Parameters 'cmap' will be ignored
  cax = scatter(
 
 
 
 
    
      


 
 
 

 
 
    
      


 
 /mnt/dzl_bioinf/exec/miniconda/miniconda3/envs/scRNA2023/lib/python3.9/site-packages/scanpy/plotting/_utils.py:430: ImplicitModificationWarning: Trying to modify attribute `._uns` of view, initializing view as actual.
  adata.uns[value_to_plot + '_colors'] = colors_list
/mnt/dzl_bioinf/exec/miniconda/miniconda3/envs/scRNA2023/lib/python3.9/site-packages/scanpy/plotting/_tools/scatterplots.py:392: UserWarning: No data for colormapping provided via 'c'. Parameters 'cmap' will be ignored
  cax = scatter(
/mnt/dzl_bioinf/exec/miniconda/miniconda3/envs/scRNA2023/lib/python3.9/site-packages/scanpy/plotting/_tools/scatterplots.py:392: UserWarning: No data for colormapping provided via 'c'. Parameters 'cmap' will be ignored
  cax = scatter(
 
 
 
 
    
      


 
 
 

 

 

 

 
 
 
 
 
  
  
 number of cells per cluster ¶  
 
 
 
  
 
 
 
 
 In [9]: 
 
      
     pd  .  set_option  (  'display.max_columns'  ,   35  ) 
 pd  .  set_option  (  'display.max_rows'  ,   None  ) 
 adata  .  obs  [  'leiden'  ]  .  value_counts  () 
 groups   =   adata  .  obs  .  groupby  ([  "leiden"  ,  "group"  ])  .  count  ()[  "sample"  ] 
 print  (  groups  ) 
  

      
 
 
 

 
 
 


 
 
    
      


 
 leiden  group      
0       NIH            3006
        Pheonix         282
        hCD34iPSC11     344
1       NIH             441
        Pheonix        2778
        hCD34iPSC11      17
2       NIH              46
        Pheonix          56
        hCD34iPSC11    1596
3       NIH            1443
        Pheonix          48
        hCD34iPSC11      53
4       NIH             112
        Pheonix         110
        hCD34iPSC11      64
Name: sample, dtype: int64
 
 
 

 

 

  
 
 
 
 
 In [10]: 
 
      
     df_counts   =   pd  .  DataFrame  (  adata  .  obs  .  groupby  ([  "leiden"  ,   "group"  ])  .  count  ()[  "batch"  ]) 
 df_counts  .  reset_index  (  inplace  =  True  ) 
 df_counts  [  "batch"  ]   =   pd  .  to_numeric  (  df_counts  [  "batch"  ]) 
 df_counts   =   df_counts  .  pivot_table  (  index  =  'leiden'  ,  columns  =  'group'  ) 
 df_counts  .  columns   =   df_counts  .  columns  .  get_level_values  (  1  ) 
 df_counts 
  

      
 
 
 

 
 
 


 
 
    
     Out[10]: 


 
 
 
 
   
     
       group 
       NIH 
       Pheonix 
       hCD34iPSC11 
     
     
       leiden 
        
        
        
     
   
   
     
       0 
       3006 
       282 
       344 
     
     
       1 
       441 
       2778 
       17 
     
     
       2 
       46 
       56 
       1596 
     
     
       3 
       1443 
       48 
       53 
     
     
       4 
       112 
       110 
       64 
     
   
 
 
 

 

 

 

  
 
 
 
 
 In [11]: 
 
      
     import   seaborn   as   sns 
 import   matplotlib.patches   as   mpatches 
 # from raw value to percentage 
 total   =    [  i  +  j  +  k   for   i  ,  j  ,  k   in   zip  (  df_counts  [  'NIH'  ],   df_counts  [  'Pheonix'  ],   df_counts  [  'hCD34iPSC11'  ])] 
 alum_bar   =   [  i   /   j   *   100   for   i  ,  j   in   zip  (  df_counts  [  'NIH'  ],   total  )] 
 ova_bar   =   [  i   /   j   *   100   for   i  ,  j   in   zip  (  df_counts  [  'Pheonix'  ],   total  )] 
 tol_bar   =   [  i   /   j   *   100   for   i  ,  j   in   zip  (  df_counts  [  'hCD34iPSC11'  ],   total  )] 

 # plot 
 barWidth   =   0.85 
 names   =   list  (  df_counts  .  index  ) 
 # Create green Bars 
 plt  .  bar  (  list  (  range  (  0  ,   len  (  names  ))),   alum_bar  ,   color  =  '#b5ffb9'  ,   edgecolor  =  'white'  ,   width  =  barWidth  ,   label  =  "NIH"  ) 
 # Create orange Bars 
 plt  .  bar  (  list  (  range  (  0  ,   len  (  names  ))),   ova_bar  ,   bottom  =  alum_bar  ,   color  =  '#f9bc86'  , 
         edgecolor  =  'white'  ,   width  =  barWidth  ,   label  =  "Pheonix"  ) 
 # Create blue Bars 
 plt  .  bar  (  list  (  range  (  0  ,   len  (  names  ))),   tol_bar  ,   bottom  =  [  i  +  j   for   i  ,  j   in   zip  (  alum_bar  ,   ova_bar  )],  
         color  =  '#a3acff'  ,   edgecolor  =  'white'  ,   width  =  barWidth  ,   label  =  "hCD34iPSC11"  ) 
 
 # Custom x axis 
 plt  .  xticks  (  list  (  range  (  0  ,   len  (  names  ))),   names  ) 
 plt  .  xlabel  (  "Cluster"  ) 
 
 # Add a legend 
 plt  .  legend  (  loc  =  'upper left'  ,   bbox_to_anchor  =  (  1  ,  1  ),   ncol  =  1  ) 

 # Show graphic 
 plt  .  grid  (  visible  =  False  ) 
 plt  .  show  () 
  

      
 
 
 

 
 
 


 
 
    
      


 
 
 

 

 

 

 
 
 
 
 
  
  
 Unsupervised Markergene identification ¶  
 
 
 
  
 
 
 
 
 In [12]: 
 
      
     #Calculate marker genes 
 adata  .  uns  [  'log1p'  ][  'base'  ]   =   None   # don't know why but looses this if saved..  
 sc  .  tl  .  rank_genes_groups  (  adata  ,   groupby  =  'leiden'  ) 
 sc  .  pl  .  rank_genes_groups  (  adata  ,   fontsize  =  14  ) 
  

      
 
 
 

 
 
 


 
 
    
      


 
 
 

 

 

 

  
 
 
 
 
 In [13]: 
 
      
     #show top 25 ranked genes per cluster 
 pd  .  set_option  (  'display.max_columns'  ,   35  ) 
 pd  .  DataFrame  (  adata  .  uns  [  'rank_genes_groups'  ][  'names'  ])  .  head  (  25  )  .  to_csv  (  "./results/automatic_marker_calcualtion_lessCluster.csv"  ) 
 pd  .  DataFrame  (  adata  .  uns  [  'rank_genes_groups'  ][  'names'  ])  .  head  (  25  ) 
  

      
 
 
 

 
 
 


 
 
    
     Out[13]: 


 
 
 
 
   
     
        
       0 
       1 
       2 
       3 
       4 
     
   
   
     
       0 
       RNASE1 
       APOE 
       RPS4Y1 
       TMSB4X 
       ANKUB1 
     
     
       1 
       MT-CO1 
       MTRNR2L1 
       LINC00278 
       F13A1 
       AP003086.1 
     
     
       2 
       MT-ND4 
       CD63 
       GPNMB 
       TXNIP 
       CPB2-AS1 
     
     
       3 
       MT-CO3 
       DBI 
       S100A10 
       FTL 
       HIF1A-AS3 
     
     
       4 
       MT-ATP6 
       RPS4X 
       MX1 
       TPT1 
       AC026202.2 
     
     
       5 
       RBPJ 
       RPS26 
       USP9Y 
       RPS14 
       AC023051.1 
     
     
       6 
       MT-ND5 
       B2M 
       VIM 
       SRGN 
       DIAPH2-AS1 
     
     
       7 
       MT-ND1 
       IGSF6 
       GNAS 
       FUCA1 
       AL136171.2 
     
     
       8 
       AL157778.1 
       CHCHD2 
       UTY 
       RBPJ 
       AL390774.2 
     
     
       9 
       MT-CO2 
       MGST3 
       HLA-B 
       RPL13 
       AC027018.1 
     
     
       10 
       MT-ND2 
       FCER1G 
       TTTY14 
       RPS18 
       TMLHE-AS1 
     
     
       11 
       TXNIP 
       ATP6AP2 
       MMP9 
       RPS8 
       EYS 
     
     
       12 
       COLEC12 
       SMS 
       IFI6 
       RPS23 
       AC024145.1 
     
     
       13 
       F13A1 
       ITM2B 
       FTH1 
       RPS4X 
       AC002463.1 
     
     
       14 
       MT-ND4L 
       GYPC 
       AP1B1 
       RPL18 
       AC087273.2 
     
     
       15 
       MTRNR2L12 
       PRDX4 
       CTSB 
       RPS3A 
       SHOC1 
     
     
       16 
       MTRNR2L8 
       CHCHD6 
       DDX3Y 
       RPL27 
       AC009554.1 
     
     
       17 
       EPS15 
       PEBP1 
       SNHG5 
       RPL7A 
       SCN1A-AS1 
     
     
       18 
       PKIB 
       RPLP1 
       SQSTM1 
       RPS13 
       KCNQ1OT1 
     
     
       19 
       PRKCA 
       RNASEK 
       PLIN2 
       RPL7 
       C12orf40 
     
     
       20 
       TCF12 
       NPC2 
       LGMN 
       RPL10 
       MYCBP2-AS1 
     
     
       21 
       STAB1 
       NACA 
       GAS6 
       RPL41 
       STARD13-AS 
     
     
       22 
       EDA 
       PRDX1 
       PRKY 
       RPL17 
       OPRM1 
     
     
       23 
       DIAPH2 
       LDHB 
       SQOR 
       RPS15A 
       RNF213-AS1 
     
     
       24 
       ARHGAP26 
       RPS2 
       MFHAS1 
       RPL29 
       AP001011.1 
     
   
 
 
 

 

 

 

  
 
 
 
 
 In [14]: 
 
      
     #Export calculated Markers (DGE) 
 result   =   adata  .  uns  [  'rank_genes_groups'  ] 
 cluster   =   result  [  'names'  ]  .  dtype  .  names 
 result 
 for   c   in   cluster  : 
     df   =   pd  .  DataFrame  ({  key  :   result  [  key  ][  c  ] 
     for   key   in   [  'names'  ,  'logfoldchanges'  ,  'pvals'  ,  'pvals_adj'  ]}) 
     df  .  to_csv  (  './results/Markergenes_combined_'  +  c  +  ".csv"  ) 
  

      
 
 
 

 
 
 
 
 
  
  
 heatmap with top 5 genes ¶  
 
 
 
  
 
 
 
 
 In [15]: 
 
      
     sc  .  pl  .  heatmap  (  adata  ,  
               np  .  unique  (  pd  .  DataFrame  (  adata  .  uns  [  'rank_genes_groups'  ][  'names'  ])  .  head  (  5  )  .  stack  ()  .  tolist  ()), 
               groupby  =  'leiden'  ,   show_gene_labels  =  True  ,   swap_axes  =  True  ,  
               figsize   =   (  55  ,  45  ),  cmap   =  "Spectral_r"  ,   save  =  "_identification_top5genes_combined.png"  ) 
  

      
 
 
 

 
 
 


 
 
    
      


 
 
 

 

 

 

  
 
 
 
 
 In [16]: 
 
      
     sc  .  pl  .  heatmap  (  adata  ,  
               np  .  unique  (  pd  .  DataFrame  (  adata  .  uns  [  'rank_genes_groups'  ][  'names'  ])  .  head  (  20  )  .  stack  ()  .  tolist  ()), 
               groupby  =  'leiden'  ,   show_gene_labels  =  True  ,   swap_axes  =  True  ,  
               figsize   =   (  55  ,  45  ),  cmap   =  "Spectral_r"  ,   save  =  "_identification_top20genes_combined.png"  ) 
  

      
 
 
 

 
 
 


 
 
    
      


 
 
 

 

 

 

 
 
 
 
 
  
  
 UMAPs of top5 genes ¶  
 
 
 
  
 
 
 
 
 In [7]: 
 
      
     sc  .  pl  .  umap  (  adata  ,  
            color  =   np  .  unique  (  pd  .  DataFrame  (  adata  .  uns  [  'rank_genes_groups'  ][  'names'  ])  .  head  (  5  )  .  stack  ()  .  tolist  ()), 
            ncols  =  2  ,   use_raw  =  True  ,   cmap  =   "RdYlGn_r"  )  #"RdYlGn") 
  

      
 
 
 

 
 
 


 
 
    
      


 
 
 

 

 

 

  
 
 
 
 
 In [5]: 
 
      
     sc  .  pl  .  umap  (  adata  ,  
            color  =   [  "STAB1"  ], 
            ncols  =  2  ,   use_raw  =  True  ,   cmap  =   "RdYlGn_r"  )  #"RdYlGn") 
  

      
 
 
 

 
 
 


 
 
    
      


 
 
 

 

 

 

  
 
 
 
 
 In [18]: 
 
      
     #[x for x in adata.var_names if x.startswith('TREM')]#Il12r 
  

      
 
 
 

  
 
 
 
 
 In [19]: 
 
      
     #[i for i in adata.var.index if "GM-" in i]  
  

      
 
 
 

 
 
 
 
 
  
  
 Heatmap of top 5 genes per cluster ¶  
 
 
 
  
 
 
 
 
 In [20]: 
 
      
     sc  .  pl  .  heatmap  (  adata  ,           
              np  .  unique  (  pd  .  DataFrame  (  adata  .  uns  [  'rank_genes_groups'  ][  'names'  ])  .  head  (  5  )  .  stack  ()  .  tolist  ()), 
            groupby  =  "leiden"  ,   log  =  True  ) 
  

      
 
 
 

 
 
 


 
 
    
      


 
 
 

 

 

 

 
 
 
 
 
  
  
 Heatmap of known genes ¶  
 
 
 
  
 
 
 
 
 In [21]: 
 
      
     genes   =   [  "TIMD4"  ,   "PLOD2"  ,   "AFP"  ,   "PARD3"  ,   "KLK8"  ,   "GAP43"  ,   "TMEM47"  ,   "PKP2"  ,   "TTR"  ,  
          "FERMT2"  ,   "TGFB2"  ,   "SERPINH1"  ,   "FGF13"  ,   "CALD1"  ,   "IGF2"  ,   "IGF2BP1"  ,   "KRT18"  ,  
          "ID1"  ,   "TPM2"  ,   "HPR"  ,   "LIN28B"  ,   "NID1"  ,   "LYVE1"  ,   "RBP4"  ,   "FLT1"  ,   "ITM2A"  ,   "MARCO"  ] 

 sc  .  pl  .  heatmap  (  adata  ,   genes  ,   groupby  =  "leiden"  ) 
 sc  .  pl  .  heatmap  (  adata  ,   genes  ,   groupby  =  [  "group"  ,   "leiden"  ],  swap_axes  =  True  ) 
  

      
 
 
 

 
 
 


 
 
    
      


 
 
 

 
 
    
      


 
 
 

 

 

 

  
 
 
 
 
 In [22]: 
 
      
     sc  .  pl  .  heatmap  (  adata  [  adata  .  obs  .  group   ==   "NIH"  ],   genes  ,   groupby  =  [  "group"  ,   "leiden"  ]) 
 sc  .  pl  .  heatmap  (  adata  [  adata  .  obs  .  group   ==   "Pheonix"  ],   genes  ,   groupby  =  [  "group"  ,   "leiden"  ]) 
 sc  .  pl  .  heatmap  (  adata  [  adata  .  obs  .  group   ==   "hCD34iPSC11"  ],   genes  ,   groupby  =  [  "group"  ,   "leiden"  ]) 
  

      
 
 
 

 
 
 


 
 
    
      


 
 
 

 
 
    
      


 
 
 

 
 
    
      


 
 
 

 

 

 

  
 
 
 
 
 In [23]: 
 
      
     genes   =   [  "FERMT2"  ,   "SERPINH1"  ,   "FGF13"  ,   "CALD1"  ,   "IGF2"  ,   "IGF2BP1"  ,  
          "LIN28B"  ,   "NID1"  ,   "LYVE1"  ] 


 Pard3 

 Fermt2 

 Serpinh1 

 Fgf13 

 Cald1 

 Igf2bp1 

 Lin28B 

 Nid1 

 Lyve1 
 sc  .  pl  .  heatmap  (  adata  ,   genes  ,   groupby  =  "leiden"  ,   save  =  "_knowngenes"  ) 
  

      
 
 
 

 
 
 


 
 
    
      


 
 
 

 

 

 

 
 
 
 
 
  
  
 Macrophage polarization states ¶  
 
 
 
  
 
 
 
 
 In [24]: 
 
      
     #[i for i in adata.var.index if "AP1" in i]  
 #[x for x in adata.var_names if x.startswith('SMAD9')] 
  

      
 
 
 

  
 
 
 
 
 In [25]: 
 
      
     markers  =   { 
     "M1"  :   [  "RELA"  ,   "STAT1"  ,   "STAT3"  ,   "IRF4"  ,   "HIF1A"  ,   "FOS"  ,   "JUN"  ,   "IFIT3"  ,   "EPSTI1"  ,   "IFI44L"  ,   "OAS2"  ,   "PSME2"  ],  
      #AP1 Transkriptionsfaktor = Gene FOS + JUN, NFKB p65 subunit = RELA Gene 
     "M2a"  :   [  "STAT6"  ,   "GATA3"  ,   "SOCS1"  ,   "PPARG"   ],  
     "M2b"  :   [  "STAT3"  ,   "IRF4"  ,   "NFKB1"  ], 
     "M2d (TAM)"  :   [  "IRF3"  ,   "NFKB1"  ,  "MARCO"  ,   "STAB1"  ]}   #Clever1 = Stab1 
  

      
 
 
 

  
 
 
 
 
 In [26]: 
 
      
     markers_list   =   np  .  unique  (  sum  (  list  (  markers  .  values  ()),   []))   # flatten the list of the dict into list 
  

      
 
 
 

  
 
 
 
 
 In [27]: 
 
      
     sc  .  pl  .  umap  (  adata  ,   color  =  markers_list  ) 
  

      
 
 
 

 
 
 


 
 
    
      


 
 
 

 

 

 

  
 
 
 
 
 In [28]: 
 
      
     sc  .  pl  .  dotplot  (  adata  ,   markers  ,   groupby  =  "leiden"  ) 
 sc  .  pl  .  stacked_violin  (  adata  ,   markers  ,   groupby  =  "leiden"  ,   cmap  =  "Reds"  ) 
 sc  .  pl  .  heatmap  (  adata  ,   markers  ,   groupby  =  "leiden"  ) 
  

      
 
 
 

 
 
 


 
 
    
      


 
 /mnt/dzl_bioinf/exec/miniconda/miniconda3/envs/scRNA2023/lib/python3.9/site-packages/scanpy/plotting/_dotplot.py:749: UserWarning: No data for colormapping provided via 'c'. Parameters 'cmap', 'norm' will be ignored
  dot_ax.scatter(x, y, **kwds)
 
 
 
 
    
      


 
 
 

 
 
    
      


 
 
 

 
 
    
      


 
 
 

 

 

 

  
 
 
 
 
 In [29]: 
 
      
     sc  .  pl  .  dotplot  (  adata  [  adata  .  obs  .  group   ==   "NIH"  ],   markers  ,   groupby  =  [  "group"  ,   "leiden"  ]) 
 sc  .  pl  .  heatmap  (  adata  [  adata  .  obs  .  group   ==   "NIH"  ],   markers  ,   groupby  =  [  "group"  ,   "leiden"  ]) 

 sc  .  pl  .  dotplot  (  adata  [  adata  .  obs  .  group   ==   "Pheonix"  ],   markers  ,   groupby  =  [  "group"  ,   "leiden"  ]) 
 sc  .  pl  .  heatmap  (  adata  [  adata  .  obs  .  group   ==   "Pheonix"  ],   markers  ,   groupby  =  [  "group"  ,   "leiden"  ]) 

 sc  .  pl  .  dotplot  (  adata  [  adata  .  obs  .  group   ==   "hCD34iPSC11"  ],   markers  ,   groupby  =  [  "group"  ,   "leiden"  ]) 
 sc  .  pl  .  heatmap  (  adata  [  adata  .  obs  .  group   ==   "hCD34iPSC11"  ],   markers  ,   groupby  =  [  "group"  ,   "leiden"  ]) 
  

      
 
 
 

 
 
 


 
 
    
      


 
 /mnt/dzl_bioinf/exec/miniconda/miniconda3/envs/scRNA2023/lib/python3.9/site-packages/scanpy/plotting/_dotplot.py:749: UserWarning: No data for colormapping provided via 'c'. Parameters 'cmap', 'norm' will be ignored
  dot_ax.scatter(x, y, **kwds)
 
 
 
 
    
      


 
 
 

 
 
    
      


 
 
 

 
 
    
      


 
 /mnt/dzl_bioinf/exec/miniconda/miniconda3/envs/scRNA2023/lib/python3.9/site-packages/scanpy/plotting/_dotplot.py:749: UserWarning: No data for colormapping provided via 'c'. Parameters 'cmap', 'norm' will be ignored
  dot_ax.scatter(x, y, **kwds)
 
 
 
 
    
      


 
 
 

 
 
    
      


 
 
 

 
 
    
      


 
 /mnt/dzl_bioinf/exec/miniconda/miniconda3/envs/scRNA2023/lib/python3.9/site-packages/scanpy/plotting/_dotplot.py:749: UserWarning: No data for colormapping provided via 'c'. Parameters 'cmap', 'norm' will be ignored
  dot_ax.scatter(x, y, **kwds)
 
 
 
 
    
      


 
 
 

 
 
    
      


 
 
 

 

 

 

 
 
 
 
 
  
  
 deregulated genes during iPSC-Mac polarization ¶  
 
 
 
  
 
 
 
 
 In [30]: 
 
      
     #print(*[i for i in markers["IL10_vs_control"] if i  not in adata.var.index ] ) 
  

      
 
 
 

  
 
 
 
 
 In [31]: 
 
      
     #list([i for i in adata.var.index if "HLA" in i] ) 
 #[x for x in adata.var_names if x.startswith('PPAR')] 
  

      
 
 
 

  
 
 
 
 
 In [32]: 
 
      
     markers  =   { 
     "IFNy_Fingerprint"  :[  "IDO1"  ,  "GBP5"  ,  "CXCL10"  ,  "CXCL10"  ,  "CXCL11"  ,  "GBP4"  ,  "ETV7"  ,  "TNFSF10"  ,  "TNFSF10"  ,  "HLA-DOA"  , 
                         "LAMP3"  ,  "APOL4"  ,  "GBP2"  ,  "IL15RA"  ,  "HLA-DQB1"  ,  "GBP1"  ,  "USP30-AS1"  ,  "CTLA4"  ,  "ANKRD22"  ,  "APOL3"  , 
                         "IRF1"  ,  "HLA-DQB1"  ,  "BATF2"  ,  "HAPLN3"  ,  "STAT1"  ,  "PSMB9"  ,  "CD38"  ,  "CIITA"  ,  "CALHM6"  ,  "LHFPL1"  , 
                         "SERPING1"  ,  "AIM2"  ,  "CEACAM1"  ,  "GRIN3A"  ,  "SAMD9L"  ,  "RSAD2"  ,  "HLA-DQB2"  ,  "C4B"  ,  "ISG20"  ,  "LYPD5"  , 
                         "CFH"  ,  "CFHR3"  ,  "SAMD9L"  ,  "IFIT2"  ,  "IFIT3"  ,  "NLRC5"  ,  "GIMAP7"  ,  "RTP4"  ,  "GCH1"  ,  "HLA-DQA2"  ,  "TAP1"  , 
                         "CFH"  ,  "IFITM1"  ,  "SECTM1"  ,  "EPSTI1"  ,  "HLA-DQA1"  ,  "HLA-DQB1"  ,  "HLA-DQA1"  ,  "GIMAP6"  ,  "WARS"  ,  "NOD2"  , 
                         "FRMD3"  ,  "HLA-DQA1"  ,  "IL15RA"  ,  "IFITM1"  ,  "APOL4"  ,  "SERPING1"  ,  "IL31RA"  ,  "IFIT2"  ,  "XAF1"  ,  "XAF1"  , 
                         "TAP2"  ,  "SERPING1"  ,  "IL15RA"  ,  "ASCL2"  ,  "CD72"  ,  "GJD3"  ,  "IFI35"  ,  "TAP2"  ,  "APOL6"  ,  "FRMD3"  ,  "HLA-DOB"  , 
                         "FAM241A"  ,  "IL15"  ,  "CFH"  ,  "IL32"  ,  "IFI44L"  ,  "GK"  ,  "SELL"  ,  "SLC6A12"  ,  "APOL6"  ,  "LINC00892"  ,  "LAG3"  , 
                         "HCAR3"  ,  "APOL6"  ,  "APOL2"  ,  "OAS2"  ,  "GBP3"  ,  "APOL1"  ,  "ASPHD2"  ,  "CEACAM1"  ,  "OASL"  ,  "ADGRE5"  ,  "IL32"  , 
                         "HLA-F"  ,  "OAS2"  ,  "IL27"  ,  "OAS2"  ,  "APOL4"  ,  "GCH1"  ,  "ASPHD2"  ,  "MPZL2"  ,  "CTLA4"  ,  "ERAP2"  ,  "CHRD"  , 
                         "FLT3LG"  ,  "FBXO6"  ,  "LHFPL1"  ,  "SCO2"  ,  "IL18BP"  ,  "DDX60"  ,  "MMP25"  ,  "BTN3A1"  ,  "C1R"  ,  "STAP1"  ,  "GIMAP5"  , 
                         "GPBAR1"  ,  "RHEBL1"  ,  "SMPD3"  ,  "OAS3"  ,  "ATP13A2"  ,  "TNF"  ,  "PSME2"  ,  "FUT1"  ,  "TRPM3"  ], 
     #not found: 'CXCL9', 'UBD', 'RARRES3', 'Sep04', 'TBX21', 'lnc-LGALS14-1', 'CXCR2P1', 'GBP1P1' 
     "IL4_Fingerprint"  :   [  "CCL26"  ,  "CCL18"  ,  "RAMP1"  ,  "CCDC85A"  ,  "CCL23"  ,  "CCDC85A"  ,  "RAMP1"  ,  "CCL17"  ,  "SUCNR1"  , 
                         "CCL13"  ,  "ZNF365"  ,  "TMEM45B"  ,  "CD209"  ,  "CISH"  ,  "P2RY12"  ,  "CLEC19A"  ,  "PCSK5"  ,  "MAOA"  ,  "PCSK5"  , 
                         "CTSC"  ,  "DACT1"  ,  "DNASE1L3"  ,  "P2RY13"  ,  "PCSK5"  ,  "F13A1"  ,  "CLEC4A"  ,  "TRIM67"  , 
                         "CD209"  ,  "CD200R1"  ,  "PPP1R14A"  ,  "CLEC4G"  ,  "CD1B"  ,  "ALOX15"  ,  "MAP1LC3C"  ,  "CD200R1"  ,  "FFAR3"  , 
                         "AP2A2"  ,  "CCL22"  ,  "CKB"  ,  "CD1C"  ,  "CD209"  ,  "HOPX"  ,  "CA4"  ,  "SLAMF1"  , 
                         "PYROXD2"  ,  "MAT2A"  ,  "HRH1"  ,  "PLXDC1"  ],  
     #not found:  PRSS40B XLOC_l2_000384 XLOC_l2_006196 HSD3B1 
     "IL10_Fingerprint"  :   [  "GAL3ST4"  ,  "CX3CR1"  ,  "S100A8"  ,  "RNASE2"  ,  "TNFSF14"  ,  "RNASE2"  ,  "APOC2"  ,  "C3"  ,  "TMEM163"  ,  "ABCG4"  , 
                          "C3"  ,  "MYLK2"  ,  "PROC"  ,  "PADI2"  ,  "SORL1"  ,  "PROC"  ,  "CAMP"  ], 
     #not found: FOXS1 
     "IFNy_vs_control"  :   [  "IDO1"  ,  "GBP5"  ,  "CXCL10"  ,  "CXCL11"  ,  "P2RY14"  ,  "GBP4"  ,  "ETV7"  , 
                         "TNFSF10"  ,  "HLA-DOA"  ,  "LAMP3"  ,  "APOL4"  ,  "GBP2"  ,  "IL15RA"  ,  "HLA-DQB1"  ,  "GBP1"  ,  "USP30-AS1"  , 
                         "CTLA4"  ,  "ANKRD22"  ,  "APOL3"  ,  "SOCS1"  ,  "IRF1"  ,  "BATF2"  ,  "HAPLN3"  ,  "STAT1"  ,  "PSMB9"  ,  "CD38"  ,  "CIITA"  , 
                         "CALHM6"  ,  "LHFPL1"  ,  "SERPING1"  ,  "AIM2"  ,  "CEACAM1"  ,  "GRIN3A"  ,  "SAMD9L"  ,  "RSAD2"  ,  "HLA-DQB2"  , 
                         "C4B"  ,  "ISG20"  ,  "LYPD5"  ,  "CFH"  ,  "CFHR3"  ,  "IFIT2"  ,  "IFIT3"  ,  "NLRC5"  ,  "GIMAP7"  ,  "RTP4"  ,  "GCH1"  ,  "HLA-DQA2"  , 
                         "TAP1"  ,  "IFITM1"  ,  "SECTM1"  ,  "EPSTI1"  ,  "HLA-DQA1"  ,  "GIMAP6"  ,  "FCGR1B"  ,  "WARS"  ,  "NOD2"  ,  "FRMD3"  ,  "IL31RA"  , 
                         "XAF1"  ,  "GPR183"  ,  "TAP2"  ,  "CD274"  ,  "ASCL2"  ,  "ALK"  ,  "CD72"  ,  "GJD3"  ,  "IFI35"  ,  "HLA-DPA1"  , 
                         "DNASE1L3"  ,  "IL21R"  ,  "APOL6"  ,  "CD163L1"  ,  "HLA-DOB"  ,  "FAM241A"  ,  "CYP27A1"  ,  "HTR2B"  ,  "IL15"  ,  "HLA-DRA"  , 
                         "SIDT1"  ,  "ABCC3"  ,  "IL32"  ,  "DEFB124"  ,  "PHACTR1"  ,  "IL2RA"  ,  "PKD2L1"  ,  "HLA-DRB5"  ,  "IFI44L"  ,  "GK"  ,  "SELL"  , 
                         "FGL2"  ,  "ERC2"  ,  "SLC6A12"  ,  "LINC00892"  ,  "VNN1"  ,  "LAG3"  ,  "SLC8A1"  ,  "HLA-DPB1"  ,  "HCAR3"  ,  "APOL2"  , 
                         "LAP3"  ,  "OAS2"  ,  "GBP3"  ,  "STAMBPL1"  ,  "APOL1"  ,  "ASPHD2"  , 
                         "OASL"  ,  "LPL"  ,  "ADGRE5"  ,  "HLA-F"  ,  "IL27"  ,  "COCH"  ,  "HLA-DRB1"  , 
                         "CD9"  ,  "PDCD1LG2"  ,  "CHIT1"  ,  "OLIG1"  ,  "CD69"  ,  "IL3RA"  ,  "SIGLEC15"  ,  "MPZL2"  ,  "OR8G5"  ,  "HAMP"  , 
                         "MANSC4"  ,  "IRF7"  ,  "TMEM71"  ,  "ERAP2"  ,  "CHRD"  ,  "CD22"  ,  "FLT3LG"  ,  "CXCR4"  ,  "ATP6V0D2"  ,  "PARP9"  ,  "FBXO6"  , 
                         "CXCL8"  ,  "ARMH1"  ,  "ALKAL2"  ,  "LINC01315"  ,  "RGCC"  ,  "SCO2"  ,  "SGPP2"  ,  "TAGAP"  ,  "IL18BP"  ,  "CD226"  ,  "DDX60"  , 
                         "PDE2A"  ,  "IL1RN"  ,  "GIMAP1"  ,  "KCNJ2"  ,  "PIM1"  ,  "ATP10A"  ,  "GIMAP4"  ,  "APOBEC3G"  ,  "SNX20"  ,  "KLHL13"  ,  "USP18"  , 
                         "MMP25"  ,  "JAK2"  ,  "BTN3A1"  ,  "SLC6A7"  ,  "C1R"  ,  "STAP1"  ,  "GIMAP5"  ,  "HPSE"  ,  "FCGR3A"  ,  "GPBAR1"  ,  "LPCAT2"  , 
                         "RHEBL1"  ,  "CD74"  ,  "SMPD3"  ,  "RTN4R"  ,  "MARCO"  ,  "CMPK2"  ,  "GFRA2"  ,  "DNASE2B"  ,  "OAS3"  ,  "BTN3A2"  , 
                         "ATP13A2"  ,  "TSPAN10"  ,  "IFI27"  ,  "TNF"  ,  "PSME2"  ,  "ISG15"  ,  "LRRC4"  ,  "DEXI"  ,  "TSIX"  , 
                         "UBE2L6"  ,  "GPNMB"  ,  "FUT1"  ,  "BATF3"  ,  "TRPM3"  ,  "FPR2"  ,  "GCSAM"  ], 
     #not found: CXCL9 UBD RARRES3 Sep04 GOLGA2P5 HLA-DPB2 HLA-DRB4 TBX21 lnc-LGALS14-1 CXCR2P1 HLA-DRB6 LOC645261 GBP1P1 RUNX1-IT1 IFITM4P 
     "IL4_vs_control"  :   [  "CCL26"  ,  "P2RY14"  ,  "CCL18"  ,  "RAMP1"  ,  "CCDC85A"  ,  "CCL23"  ,  "CCL17"  ,  "SOCS1"  ,  "SUCNR1"  , 
                        "CCL13"  ,  "ZNF365"  ,  "TMEM236"  ,  "TMEM45B"  ,  "CD209"  ,  "HS3ST1"  ,  "CISH"  ,  "P2RY12"  ,  "CLEC19A"  ,  "PCSK5"  , 
                        "MAOA"  ,  "FGL2"  ,  "IL1RN"  ,  "BATF3"  ,  "CTSC"  ,  "DACT1"  ,  "DNASE1L3"  ,  "P2RY13"  ,  "F13A1"  , 
                        "CLEC4A"  ,  "TRIM67"  ,  "PTGS1"  ,  "GGT5"  ,  "CD200R1"  ,  "PPP1R14A"  ,  "CLEC4G"  ,  "IPCEF1"  ,  "CD1B"  ,  "ALOX15"  , 
                        "MAP1LC3C"  ,  "FFAR3"  ,  "AP2A2"  ,  "MYCL"  ,  "CCL22"  ,  "CCL8"  ,  "TGM2"  ,  "CKB"  ,  "CD1C"  ,  "CD2"  , 
                        "ANKEF1"  ,  "CXCL8"  ,  "NFE2"  ,  "IL1B"  ,  "HOPX"  ,  "KCNJ2"  ,  "CXCL12"  ,  "SIGLEC8"  ,  "FGF13"  ,  "PLPPR4"  , 
                        "TMEM26"  ,  "SORCS1"  ,  "CA4"  ,  "SLAMF1"  ,  "CARD9"  ,  "LINC00996"  ,  "PDCD1LG2"  ,  "MMP25"  , 
                        "CCL14"  ,  "CACNB4"  ,  "PYROXD2"  ,  "MIR3142HG"  ,  "MAT2A"  ,  "VNN1"  ,  "HRH1"  ,  "B3GNT5"  ,  "ALDH1A2"  ,  "GIMAP8"  , 
                        "PLXDC1"  ,  "SLA"  ,  "CHDH"  ], 
     #not found:  PRSS40B XLOC_l2_000384 XLOC_l2_006196 HSD3B1 TACSTD2 NTSR2 
     "IL10_vs_control"  :   [  "GAL3ST4"  ,  "CX3CR1"  ,  "S100A8"  ,  "CCL13"  ,  "DCANP1"  ,  "TIFAB"  ,  "RNASE2"  ,  "TNFSF14"  ,  "IL21R"  ,  "SIGLEC10"  , 
                         "PTGIR"  ,  "RHOH"  ,  "APOC2"  ,  "C3"  ,  "TMEM163"  ,  "ABCG4"  ,  "FPR1"  ,  "FCGR3A"  ,  "HLA-DOA"  ,  "MYLK2"  ,  "SLC11A1"  , 
                         "IL2RA"  ,  "PROC"  ,  "PADI2"  ,  "CLDN11"  ,  "AKR1B10"  ,  "MMP25"  ,  "RNASE3"  ,  "SORL1"  ,  "MIAT"  ,  "CAMP"  , 
                         "RIPOR3"  ,  "PALD1"  ,  "AKR1B15"  ,  "AGMAT"  ,  "HS3ST1"  ]} 
     #not found: FOXS1 
  

      
 
 
 

  
 
 
 
 
 In [33]: 
 
      
     sc  .  pl  .  heatmap  (  adata  ,   markers  ,   groupby  =  "leiden"  ) 
 sc  .  pl  .  dotplot  (  adata  ,   markers  ,   groupby  =  "leiden"  ) 
 #sc.pl.matrixplot(adata, markers, groupby="leiden") 
  

      
 
 
 

 
 
 


 
 
    
      


 
 
 

 
 
    
      


 
 /mnt/dzl_bioinf/exec/miniconda/miniconda3/envs/scRNA2023/lib/python3.9/site-packages/scanpy/plotting/_dotplot.py:749: UserWarning: No data for colormapping provided via 'c'. Parameters 'cmap', 'norm' will be ignored
  dot_ax.scatter(x, y, **kwds)
 
 
 
 
    
      


 
 
 

 

 

 

  
 
 
 
 
 In [34]: 
 
      
     for   m   in   markers  : 
     print  (  m  ) 
     sc  .  pl  .  dotplot  (  adata  ,   markers  [  m  ],   groupby  =  "leiden"  ) 
     sc  .  pl  .  heatmap  (  adata  ,   markers  [  m  ],   groupby  =  "leiden"  ) 
    
     sc  .  pl  .  dotplot  (  adata  ,   markers  [  m  ],   groupby  =  [  "group"  ,   "leiden"  ]) 
    # sc.pl.heatmap(adata, markers[m], groupby=["group", "leiden"]) 
    
     sc  .  pl  .  dotplot  (  adata  [  adata  .  obs  .  group   ==   "NIH"  ],   markers  [  m  ],   groupby  =  [  "group"  ,   "leiden"  ]) 
     #sc.pl.heatmap(adata[adata.obs.group == "NIH"], markers, groupby=["group", "leiden"]) 

     sc  .  pl  .  dotplot  (  adata  [  adata  .  obs  .  group   ==   "Pheonix"  ],   markers  [  m  ],   groupby  =  [  "group"  ,   "leiden"  ]) 
     #sc.pl.heatmap(adata[adata.obs.group == "Pheonix"], markers, groupby=["group", "leiden"]) 

     sc  .  pl  .  dotplot  (  adata  [  adata  .  obs  .  group   ==   "hCD34iPSC11"  ],   markers  [  m  ],   groupby  =  [  "group"  ,   "leiden"  ]) 
     #sc.pl.heatmap(adata[adata.obs.group == "hCD34iPSC11"], markers, groupby=["group", "leiden"]) 
  

      
 
 
 

 
 
 


 
 
    
      


 
 IFNy_Fingerprint
 
 
 
 
    
      


 
 /mnt/dzl_bioinf/exec/miniconda/miniconda3/envs/scRNA2023/lib/python3.9/site-packages/scanpy/plotting/_dotplot.py:749: UserWarning: No data for colormapping provided via 'c'. Parameters 'cmap', 'norm' will be ignored
  dot_ax.scatter(x, y, **kwds)
 
 
 
 
    
      


 
 
 

 
 
    
      


 
 
 

 
 
    
      


 
 /mnt/dzl_bioinf/exec/miniconda/miniconda3/envs/scRNA2023/lib/python3.9/site-packages/scanpy/plotting/_dotplot.py:749: UserWarning: No data for colormapping provided via 'c'. Parameters 'cmap', 'norm' will be ignored
  dot_ax.scatter(x, y, **kwds)
 
 
 
 
    
      


 
 
 

 
 
    
      


 
 /mnt/dzl_bioinf/exec/miniconda/miniconda3/envs/scRNA2023/lib/python3.9/site-packages/scanpy/plotting/_dotplot.py:749: UserWarning: No data for colormapping provided via 'c'. Parameters 'cmap', 'norm' will be ignored
  dot_ax.scatter(x, y, **kwds)
 
 
 
 
    
      


 
 
 

 
 
    
      


 
 /mnt/dzl_bioinf/exec/miniconda/miniconda3/envs/scRNA2023/lib/python3.9/site-packages/scanpy/plotting/_dotplot.py:749: UserWarning: No data for colormapping provided via 'c'. Parameters 'cmap', 'norm' will be ignored
  dot_ax.scatter(x, y, **kwds)
 
 
 
 
    
      


 
 
 

 
 
    
      


 
 /mnt/dzl_bioinf/exec/miniconda/miniconda3/envs/scRNA2023/lib/python3.9/site-packages/scanpy/plotting/_dotplot.py:749: UserWarning: No data for colormapping provided via 'c'. Parameters 'cmap', 'norm' will be ignored
  dot_ax.scatter(x, y, **kwds)
 
 
 
 
    
      


 
 
 

 
 
    
      


 
 IL4_Fingerprint
 
 
 
 
    
      


 
 /mnt/dzl_bioinf/exec/miniconda/miniconda3/envs/scRNA2023/lib/python3.9/site-packages/scanpy/plotting/_dotplot.py:749: UserWarning: No data for colormapping provided via 'c'. Parameters 'cmap', 'norm' will be ignored
  dot_ax.scatter(x, y, **kwds)
 
 
 
 
    
      


 
 
 

 
 
    
      


 
 
 

 
 
    
      


 
 /mnt/dzl_bioinf/exec/miniconda/miniconda3/envs/scRNA2023/lib/python3.9/site-packages/scanpy/plotting/_dotplot.py:749: UserWarning: No data for colormapping provided via 'c'. Parameters 'cmap', 'norm' will be ignored
  dot_ax.scatter(x, y, **kwds)
 
 
 
 
    
      


 
 
 

 
 
    
      


 
 /mnt/dzl_bioinf/exec/miniconda/miniconda3/envs/scRNA2023/lib/python3.9/site-packages/scanpy/plotting/_dotplot.py:749: UserWarning: No data for colormapping provided via 'c'. Parameters 'cmap', 'norm' will be ignored
  dot_ax.scatter(x, y, **kwds)
 
 
 
 
    
      


 
 
 

 
 
    
      


 
 /mnt/dzl_bioinf/exec/miniconda/miniconda3/envs/scRNA2023/lib/python3.9/site-packages/scanpy/plotting/_dotplot.py:749: UserWarning: No data for colormapping provided via 'c'. Parameters 'cmap', 'norm' will be ignored
  dot_ax.scatter(x, y, **kwds)
 
 
 
 
    
      


 
 
 

 
 
    
      


 
 /mnt/dzl_bioinf/exec/miniconda/miniconda3/envs/scRNA2023/lib/python3.9/site-packages/scanpy/plotting/_dotplot.py:749: UserWarning: No data for colormapping provided via 'c'. Parameters 'cmap', 'norm' will be ignored
  dot_ax.scatter(x, y, **kwds)
 
 
 
 
    
      


 
 
 

 
 
    
      


 
 IL10_Fingerprint
 
 
 
 
    
      


 
 /mnt/dzl_bioinf/exec/miniconda/miniconda3/envs/scRNA2023/lib/python3.9/site-packages/scanpy/plotting/_dotplot.py:749: UserWarning: No data for colormapping provided via 'c'. Parameters 'cmap', 'norm' will be ignored
  dot_ax.scatter(x, y, **kwds)
 
 
 
 
    
      


 
 
 

 
 
    
      


 
 
 

 
 
    
      


 
 /mnt/dzl_bioinf/exec/miniconda/miniconda3/envs/scRNA2023/lib/python3.9/site-packages/scanpy/plotting/_dotplot.py:749: UserWarning: No data for colormapping provided via 'c'. Parameters 'cmap', 'norm' will be ignored
  dot_ax.scatter(x, y, **kwds)
 
 
 
 
    
      


 
 
 

 
 
    
      


 
 /mnt/dzl_bioinf/exec/miniconda/miniconda3/envs/scRNA2023/lib/python3.9/site-packages/scanpy/plotting/_dotplot.py:749: UserWarning: No data for colormapping provided via 'c'. Parameters 'cmap', 'norm' will be ignored
  dot_ax.scatter(x, y, **kwds)
 
 
 
 
    
      


 
 
 

 
 
    
      


 
 /mnt/dzl_bioinf/exec/miniconda/miniconda3/envs/scRNA2023/lib/python3.9/site-packages/scanpy/plotting/_dotplot.py:749: UserWarning: No data for colormapping provided via 'c'. Parameters 'cmap', 'norm' will be ignored
  dot_ax.scatter(x, y, **kwds)
 
 
 
 
    
      


 
 
 

 
 
    
      


 
 /mnt/dzl_bioinf/exec/miniconda/miniconda3/envs/scRNA2023/lib/python3.9/site-packages/scanpy/plotting/_dotplot.py:749: UserWarning: No data for colormapping provided via 'c'. Parameters 'cmap', 'norm' will be ignored
  dot_ax.scatter(x, y, **kwds)
 
 
 
 
    
      


 
 
 

 
 
    
      


 
 IFNy_vs_control
 
 
 
 
    
      


 
 /mnt/dzl_bioinf/exec/miniconda/miniconda3/envs/scRNA2023/lib/python3.9/site-packages/scanpy/plotting/_dotplot.py:749: UserWarning: No data for colormapping provided via 'c'. Parameters 'cmap', 'norm' will be ignored
  dot_ax.scatter(x, y, **kwds)
 
 
 
 
    
      


 
 
 

 
 
    
      


 
 
 

 
 
    
      


 
 /mnt/dzl_bioinf/exec/miniconda/miniconda3/envs/scRNA2023/lib/python3.9/site-packages/scanpy/plotting/_dotplot.py:749: UserWarning: No data for colormapping provided via 'c'. Parameters 'cmap', 'norm' will be ignored
  dot_ax.scatter(x, y, **kwds)
 
 
 
 
    
      


 
 
 

 
 
    
      


 
 /mnt/dzl_bioinf/exec/miniconda/miniconda3/envs/scRNA2023/lib/python3.9/site-packages/scanpy/plotting/_dotplot.py:749: UserWarning: No data for colormapping provided via 'c'. Parameters 'cmap', 'norm' will be ignored
  dot_ax.scatter(x, y, **kwds)
 
 
 
 
    
      


 
 
 

 
 
    
      


 
 /mnt/dzl_bioinf/exec/miniconda/miniconda3/envs/scRNA2023/lib/python3.9/site-packages/scanpy/plotting/_dotplot.py:749: UserWarning: No data for colormapping provided via 'c'. Parameters 'cmap', 'norm' will be ignored
  dot_ax.scatter(x, y, **kwds)
 
 
 
 
    
      


 
 
 

 
 
    
      


 
 /mnt/dzl_bioinf/exec/miniconda/miniconda3/envs/scRNA2023/lib/python3.9/site-packages/scanpy/plotting/_dotplot.py:749: UserWarning: No data for colormapping provided via 'c'. Parameters 'cmap', 'norm' will be ignored
  dot_ax.scatter(x, y, **kwds)
 
 
 
 
    
      


 
 
 

 
 
    
      


 
 IL4_vs_control
 
 
 
 
    
      


 
 /mnt/dzl_bioinf/exec/miniconda/miniconda3/envs/scRNA2023/lib/python3.9/site-packages/scanpy/plotting/_dotplot.py:749: UserWarning: No data for colormapping provided via 'c'. Parameters 'cmap', 'norm' will be ignored
  dot_ax.scatter(x, y, **kwds)
 
 
 
 
    
      


 
 
 

 
 
    
      


 
 
 

 
 
    
      


 
 /mnt/dzl_bioinf/exec/miniconda/miniconda3/envs/scRNA2023/lib/python3.9/site-packages/scanpy/plotting/_dotplot.py:749: UserWarning: No data for colormapping provided via 'c'. Parameters 'cmap', 'norm' will be ignored
  dot_ax.scatter(x, y, **kwds)
 
 
 
 
    
      


 
 
 

 
 
    
      


 
 /mnt/dzl_bioinf/exec/miniconda/miniconda3/envs/scRNA2023/lib/python3.9/site-packages/scanpy/plotting/_dotplot.py:749: UserWarning: No data for colormapping provided via 'c'. Parameters 'cmap', 'norm' will be ignored
  dot_ax.scatter(x, y, **kwds)
 
 
 
 
    
      


 
 
 

 
 
    
      


 
 /mnt/dzl_bioinf/exec/miniconda/miniconda3/envs/scRNA2023/lib/python3.9/site-packages/scanpy/plotting/_dotplot.py:749: UserWarning: No data for colormapping provided via 'c'. Parameters 'cmap', 'norm' will be ignored
  dot_ax.scatter(x, y, **kwds)
 
 
 
 
    
      


 
 
 

 
 
    
      


 
 /mnt/dzl_bioinf/exec/miniconda/miniconda3/envs/scRNA2023/lib/python3.9/site-packages/scanpy/plotting/_dotplot.py:749: UserWarning: No data for colormapping provided via 'c'. Parameters 'cmap', 'norm' will be ignored
  dot_ax.scatter(x, y, **kwds)
 
 
 
 
    
      


 
 
 

 
 
    
      


 
 IL10_vs_control
 
 
 
 
    
      


 
 /mnt/dzl_bioinf/exec/miniconda/miniconda3/envs/scRNA2023/lib/python3.9/site-packages/scanpy/plotting/_dotplot.py:749: UserWarning: No data for colormapping provided via 'c'. Parameters 'cmap', 'norm' will be ignored
  dot_ax.scatter(x, y, **kwds)
 
 
 
 
    
      


 
 
 

 
 
    
      


 
 
 

 
 
    
      


 
 /mnt/dzl_bioinf/exec/miniconda/miniconda3/envs/scRNA2023/lib/python3.9/site-packages/scanpy/plotting/_dotplot.py:749: UserWarning: No data for colormapping provided via 'c'. Parameters 'cmap', 'norm' will be ignored
  dot_ax.scatter(x, y, **kwds)
 
 
 
 
    
      


 
 
 

 
 
    
      


 
 /mnt/dzl_bioinf/exec/miniconda/miniconda3/envs/scRNA2023/lib/python3.9/site-packages/scanpy/plotting/_dotplot.py:749: UserWarning: No data for colormapping provided via 'c'. Parameters 'cmap', 'norm' will be ignored
  dot_ax.scatter(x, y, **kwds)
 
 
 
 
    
      


 
 
 

 
 
    
      


 
 /mnt/dzl_bioinf/exec/miniconda/miniconda3/envs/scRNA2023/lib/python3.9/site-packages/scanpy/plotting/_dotplot.py:749: UserWarning: No data for colormapping provided via 'c'. Parameters 'cmap', 'norm' will be ignored
  dot_ax.scatter(x, y, **kwds)
 
 
 
 
    
      


 
 
 

 
 
    
      


 
 /mnt/dzl_bioinf/exec/miniconda/miniconda3/envs/scRNA2023/lib/python3.9/site-packages/scanpy/plotting/_dotplot.py:749: UserWarning: No data for colormapping provided via 'c'. Parameters 'cmap', 'norm' will be ignored
  dot_ax.scatter(x, y, **kwds)
 
 
 
 
    
      


 
 
 

 

 

 

  
 
 
 
 
 In [35]: 
 
      
     markers  =   [  "STAT1"  ,  "EPSTI1"  ,  "IFI44L"  ,  "OAS2"  ,  "OAS2"  ,  "OAS3"  ,  "PSME2"  ] 

 sc  .  pl  .  dotplot  (  adata  ,   markers  ,   groupby  =  "leiden"  ) 
  

      
 
 
 

 
 
 


 
 
    
      


 
 /mnt/dzl_bioinf/exec/miniconda/miniconda3/envs/scRNA2023/lib/python3.9/site-packages/scanpy/plotting/_dotplot.py:749: UserWarning: No data for colormapping provided via 'c'. Parameters 'cmap', 'norm' will be ignored
  dot_ax.scatter(x, y, **kwds)
 
 
 
 
    
      


 
 
 

 

 

 

 
 
 
 
 
  
  
 Conserved lineage macrophages ¶  
 
 
 
  
 
 
 
 
 In [36]: 
 
      
     markers_lineage   =   [  "TMEM273"  ,  "ABHD12"  ,  "ACP2"  ,  "AGMO"  ,  "AKR1B10"  ,  "ASPH"  ,  "BAG3"  ,  "BEND6"  ,  "CAMK1"  ,  "CD14"  , 
                    "CD302"  ,  "CNDP2"  ,  "COMT"  ,  "COPZ2"  ,  "CTNND1"  ,  "CTSB"  ,  "CTSF"  ,  "CTSL"  ,  "DHRS3"  , 
                    "DOCK1"  ,  "DPP7"  ,  "EXTL2"  ,  "FBLIM1"  ,  "FRRS1"  ,  "GABARAPL1"  ,  "GBGT1"  , 
                    "GGH"  ,  "HSPA13"  ,  "IL1A"  ,  "JUP"  ,  "LEPROT"  ,  "LIPA"  ,  "LYPLAL1"  , 
                    "MAN1C1"  ,  "MERTK"  ,  "MITF"  ,  "NRP1"  ,  "NXT2"  ,  "PARVB"  ,  "PCYOX1"  ,  "PECR"  ,  "PKD2"  , 
                    "PLA2G15"  ,  "PLOD1"  ,  "RENBP"  ,  "SCARB2"  ,  "SLC16A7"  ,  "SLC7A7"  ,  "SLC7A8"  ,  "SNX7"  , 
                    "SRXN1"  ,  "TMBIM1"  ,  "TMCC3"  ,  "TMEM106A"  ,  "TMEM159"  ,  "TPP1"  ,  "WLS"  ,  "ZEB2"  ] 
  

      
 
 
 

  
 
 
 
 
 In [37]: 
 
      
     sc  .  pl  .  heatmap  (  adata  ,   markers_lineage  ,   groupby  =  "leiden"  ) 
 sc  .  pl  .  dotplot  (  adata  ,   markers_lineage  ,   groupby  =  "leiden"  ) 
 #sc.pl.matrixplot(adata, markers, groupby="leiden") 
  

      
 
 
 

 
 
 


 
 
    
      


 
 
 

 
 
    
      


 
 /mnt/dzl_bioinf/exec/miniconda/miniconda3/envs/scRNA2023/lib/python3.9/site-packages/scanpy/plotting/_dotplot.py:749: UserWarning: No data for colormapping provided via 'c'. Parameters 'cmap', 'norm' will be ignored
  dot_ax.scatter(x, y, **kwds)
 
 
 
 
    
      


 
 
 

 

 

 

  
 
 
 
 
 In [38]: 
 
      
     #subset 
 markers_lineage   =   [  "ABHD12"  ,  "ASPH"  ,  "CD14"  , 
                    "CNDP2"  ,  "CTNND1"  ,  "CTSB"  ,  "CTSL"  ,  "DPP7"  ,  "LEPROT"  ,  "LIPA"  ,  "LYPLAL1"  , 
                    "MAN1C1"  ,  "MERTK"  ,  "MITF"  ,  "NRP1"  ,  "SCARB2"  ,  "SLC7A7"  ,  "ZEB2"  ] 

 #sc.pl.heatmap(adata, markers_lineage, groupby="leiden") 
 sc  .  pl  .  dotplot  (  adata  ,   markers_lineage  ,   groupby  =  "leiden"  ) 
  

      
 
 
 

 
 
 


 
 
    
      


 
 /mnt/dzl_bioinf/exec/miniconda/miniconda3/envs/scRNA2023/lib/python3.9/site-packages/scanpy/plotting/_dotplot.py:749: UserWarning: No data for colormapping provided via 'c'. Parameters 'cmap', 'norm' will be ignored
  dot_ax.scatter(x, y, **kwds)
 
 
 
 
    
      


 
 
 

 

 

 

 
 
 
 
 
  
  
 UMAP CD11b, CD14, CD163, CD86 ¶  
 
 
 
  
 
 
 
 
 In [39]: 
 
      
     #[i for i in adata.var.index if "AP1" in i]  
 #[x for x in adata.var_names if x.startswith('HLA')] 
  

      
 
 
 

  
 
 
 
 
 In [40]: 
 
      
     sc  .  pl  .  umap  (  adata  ,   color  =  [  "ITGAM"  ,   "CD14"  ,  "CD163"  ,  "CD86"  ,  "MRC1"  ,  "FCGR1A"  ,  "FCGR3A"  ,  'HLA-DRA'  ],   ncols  =  2  ) 
  

      
 
 
 

 
 
 


 
 
    
      


 
 
 

 

 

 

  
 
 
 
 
 In [41]: 
 
      
     sc  .  pl  .  violin  (  adata  ,   keys  =  [  "ITGAM"  ,   "CD14"  ,  "CD163"  ,  "CD86"  ], 
              groupby  =  'group'  ,   rotation  =  90   ,   save  =  "_genes1"  ) 

 sc  .  pl  .  violin  (  adata  ,   keys  =  [  "MRC1"  ,  "FCGR1A"  ,  "FCGR3A"  ,  'HLA-DRA'  ], 
              groupby  =  'group'  ,   rotation  =  90  ,   save  =  "_genes2"  ) 
  

      
 
 
 

 
 
 


 
 
    
      


 
 
 

 
 
    
      


 
 
 

 

 

 

  
 
 
 
 
 In [42]: 
 
      
     sc  .  pl  .  violin  (  adata  ,   keys  =  [  "ITGAM"  ,   "CD14"  ,  "CD163"  ,  "CD86"  ], 
              groupby  =  'leiden'  ,   rotation  =  90  ,   save  =  "_genes1_clusters"  ) 

 sc  .  pl  .  violin  (  adata  ,   keys  =  [  "MRC1"  ,  "FCGR1A"  ,  "FCGR3A"  ,  'HLA-DRA'  ], 
              groupby  =  'leiden'  ,   rotation  =  90  ,   save  =  "_genes2_clusters"  ) 
  

      
 
 
 

 
 
 


 
 
    
      


 
 
 

 
 
    
      


 
 
 

 

 

 

 
 
 
 
 
  
  
 Save results ¶  
 
 
 
  
 
 
 
 
 In [43]: 
 
      
     print  (  './results/CelltypeIdentifcation_202401_lessClusters.h5ad'   ) 
 #adata.write('./results/CelltypeIdentifcation_202401_lessClusters.h5ad' ) 
  

      
 
 
 

 
 
 


 
 
    
      


 
 ./results/CelltypeIdentifcation_202401_lessClusters.h5ad
 
 
 

 

 

  
 
 
 
 
 In [2]: 
 
      
     adata   =   sc  .  read  (  './results/CelltypeIdentifcation_202401_lessClusters.h5ad'  ) 
  

      
 
 
 

  
 
 
 
 
 In [8]: 
 
      
    %%bash
jupyter   nbconvert   --to    html_toc   CelltypeIdentifcation_202401_lessClusters.ipynb   --ExtractOutputPreprocessor.enabled = False
mv   CelltypeIdentifcation_202401_lessClusters.html   ./results/CelltypeIdentifcation_202401_lessClusters_ $( date   +%Y%m%d ) .html
  

      
 
 
 

 
 
 


 
 
    
      


 
 [NbConvertApp] WARNING | Config option `kernel_spec_manager_class` not recognized by `NbConvertApp`.
[NbConvertApp] Converting notebook CelltypeIdentifcation_202401_lessClusters.ipynb to html_toc
[NbConvertApp] Writing 53501977 bytes to CelltypeIdentifcation_202401_lessClusters.html
 
 
 

 

 

 
 


 
