# Supplementary material for: Standardized generation of human iPSC-derived hematopoietic organoids and macrophages utilizing a benchtop bioreactor platform under fully defined conditions
Source: Stem Cell Res Ther. 2024 Jun 18;15:171. doi: 10.1186/s13287-024-03785-2 (PMC11184717; doi:10.1186/s13287-024-03785-2)
Supplement: Supplementary file 8 — Additional file 8. [file 13287_2024_3785_MOESM8_ESM.html]

None


Velocity\_analysis


In [1]:

```
%%bash
echo "Current Conda environment: ${CONDA_DEFAULT_ENV}"
```

```
Current Conda environment: scRNA2023
```

In [2]:

```
%matplotlib inline
#%load_ext memory_profiler
import sys
sys.path.append("/mnt/dzl_bioinf/exec/miniconda/miniconda3/envs/scRNA/lib/python3.7/site-packages'")
import os
import numpy as np
import pandas as pd
import scanpy as sc
import scvelo as scv
import datetime
import scipy
import matplotlib.pyplot as plt

os.chdir("/mnt/dzl_bioinf/gaedckes/scRNASeq/Macrophagen_AGLachmann_202311/")

sc.settings.verbosity = 0         # verbosity: errors (0), warnings (1), info (2), hints (3)
sc.logging.print_versions()
sc.set_figure_params(scanpy=True, dpi=100, dpi_save=150,
                         frameon=True, vector_friendly=True, 
                         fontsize=14, color_map=None, format='pdf',
                         transparent=True )
sc._settings.n_jobs = 5
sc._settings.max_memory = 80 #GB
plt.rcParams['figure.figsize']=(5,5) #rescale figures
sc._settings.savefigs = True
```

```
-----
anndata     0.7.8
scanpy      1.9.3
-----
Cython                      3.0.2
PIL                         9.4.0
absl                        NA
aiohttp                     3.8.3
aiosignal                   1.2.0
annotated_types             0.5.0
anyio                       NA
asciitree                   NA
asttokens                   NA
async_timeout               4.0.2
attr                        22.1.0
backcall                    0.2.0
backoff                     2.2.1
beta_ufunc                  NA
binom_ufunc                 NA
bottleneck                  1.3.5
brotli                      NA
bs4                         4.12.2
certifi                     2023.07.22
cffi                        1.15.1
charset_normalizer          2.0.4
chex                        0.1.7
click                       8.1.7
cloudpickle                 2.2.1
colorama                    0.4.6
comm                        0.1.2
contextlib2                 NA
croniter                    NA
cycler                      0.10.0
cython                      3.0.2
cython_runtime              NA
cytoolz                     0.12.0
dask                        2023.4.1
dateutil                    2.8.2
debugpy                     1.5.1
decorator                   5.1.1
deepdiff                    6.5.0
defusedxml                  0.7.1
docrep                      0.3.2
entrypoints                 0.4
etils                       1.4.1
executing                   0.8.3
fastapi                     0.103.1
fasteners                   NA
flax                        0.7.3
frozenlist                  1.3.3
fsspec                      2023.4.0
h5py                        3.1.0
hypergeom_ufunc             NA
idna                        3.4
igraph                      0.9.10
importlib_resources         NA
ipykernel                   6.19.2
ipython_genutils            0.2.0
ipywidgets                  8.0.4
jax                         0.4.14
jaxlib                      0.4.14
jedi                        0.18.1
jinja2                      3.1.2
joblib                      1.2.0
kiwisolver                  1.4.4
leidenalg                   0.8.10
lightning                   2.0.8
lightning_cloud             NA
lightning_utilities         0.9.0
llvmlite                    0.40.0
lxml                        4.9.2
markupsafe                  2.1.1
matplotlib                  3.7.1
matplotlib_inline           0.1.6
ml_collections              NA
ml_dtypes                   0.2.0
mpl_toolkits                NA
mpmath                      1.3.0
msgpack                     1.0.3
mudata                      0.2.3
multidict                   6.0.2
multipart                   0.0.6
multipledispatch            0.6.0
natsort                     7.1.1
nbinom_ufunc                NA
ncf_ufunc                   NA
numba                       0.57.1
numcodecs                   0.11.0
numexpr                     2.8.4
numpy                       1.24.4
numpyro                     0.13.0
nvfuser                     NA
opt_einsum                  v3.3.0
optax                       0.1.7
ordered_set                 4.1.0
packaging                   23.0
pandas                      1.5.3
parso                       0.8.3
pexpect                     4.8.0
pickleshare                 0.7.5
pkg_resources               NA
platformdirs                2.5.2
prompt_toolkit              3.0.36
psutil                      5.9.0
ptyprocess                  0.7.0
pure_eval                   0.2.2
pydantic                    2.1.1
pydantic_core               2.4.0
pydev_ipython               NA
pydevconsole                NA
pydevd                      2.6.0
pydevd_concurrency_analyser NA
pydevd_file_utils           NA
pydevd_plugins              NA
pydevd_tracing              NA
pygments                    2.15.1
pyparsing                   3.0.9
pyro                        1.8.6
pytz                        2022.7
requests                    2.29.0
rich                        NA
scipy                       1.9.3
scvelo                      0.3.0
scvi                        1.0.3
session_info                1.0.0
setuptools                  67.8.0
six                         1.16.0
sklearn                     1.1.3
sniffio                     1.2.0
socks                       1.7.1
soupsieve                   2.4
sparse                      0.14.0
sphinxcontrib               NA
stack_data                  0.2.0
starlette                   0.27.0
sympy                       1.12
tblib                       1.7.0
texttable                   1.6.4
threadpoolctl               2.2.0
tlz                         0.12.0
toolz                       0.12.0
torch                       2.0.1+cu117
torchmetrics                1.1.2
tornado                     6.2
tqdm                        4.66.1
traitlets                   5.7.1
tree                        0.1.8
typing_extensions           NA
urllib3                     1.26.15
uvicorn                     0.23.2
wcwidth                     0.2.5
websocket                   0.58.0
websockets                  11.0.3
wrapt                       1.14.1
xarray                      2023.8.0
yaml                        6.0
yarl                        1.8.1
zarr                        2.13.3
zipp                        NA
zmq                         25.0.2
zoneinfo                    NA
-----
IPython             8.12.0
jupyter_client      8.1.0
jupyter_core        5.3.0
notebook            6.5.4
-----
Python 3.9.16 (main, Mar  8 2023, 14:00:05) [GCC 11.2.0]
Linux-5.4.0-122-generic-x86_64-with-glibc2.31
-----
Session information updated at 2024-02-06 14:36
```

# Load loom file¶

is created in velocity.ipynb

In [3]:

```
import loompy
loom_path="/mnt/dzl_bioinf/gaedckes/scRNASeq/Macrophagen_AGLachmann_202311/velocity/"
ldata_34 = sc.read_loom(loom_path+"N4934/N4934_possorted_genome_bam_2OGWC.loom",  validate=False)
ldata_35 = sc.read_loom(loom_path+"N4935/N4935_possorted_genome_bam_02VGP.loom",  validate=False)
ldata_36 = sc.read_loom(loom_path+"N4936/N4936_possorted_genome_bam_V4AXR.loom",  validate=False)

ldata_34.var["sample"] = "N4934"
ldata_35.var["sample"] = "N4935"
ldata_36.var["sample"] = "N4936"

ldata_34.write_loom(loom_path+"N4934/N4934_possorted_genome_bam_2OGWC_prepro.loom")
ldata_35.write_loom(loom_path+"N4935/N4935_possorted_genome_bam_02VGP_prepro.loom")
ldata_36.write_loom(loom_path+"N4936/N4936_possorted_genome_bam_V4AXR_prepro.loom")

files = [loom_path+"N4934/N4934_possorted_genome_bam_2OGWC_prepro.loom",
         loom_path+"N4935/N4935_possorted_genome_bam_02VGP_prepro.loom",
         loom_path+"N4936/N4936_possorted_genome_bam_V4AXR_prepro.loom"]

loompy.combine(files, output_file=loom_path+"N1729-N1730_possorted_genome_bam.loom", key="Accession")
```

```
WARNING:root:╭── 'batch_scan_layers' is deprecated. Use 'scan' instead
WARNING:root:╰──> at /mnt/dzl_bioinf/exec/miniconda/miniconda3/envs/scRNA2023/lib/python3.9/site-packages/loompy/loompy.py, line 471
WARNING:root:╭── 'batch_scan_layers' is deprecated. Use 'scan' instead
WARNING:root:╰──> at /mnt/dzl_bioinf/exec/miniconda/miniconda3/envs/scRNA2023/lib/python3.9/site-packages/loompy/loompy.py, line 471
```

In [4]:

```
ldata = sc.read_loom(loom_path+"N1729-N1730_possorted_genome_bam.loom",  validate=False)
ldata.var_names_make_unique()
ldata
```

Out[4]:

```
AnnData object with n_obs × n_vars = 10831 × 36601
    var: 'Accession', 'Chromosome', 'End', 'Start', 'Strand', 'sample'
    layers: 'matrix', 'ambiguous', 'spliced', 'unspliced'
```

# Velocity analysis¶

In [5]:

```
adata = sc.read("/mnt/dzl_bioinf/gaedckes/scRNASeq/Macrophagen_AGLachmann_202311/results/CelltypeIdentifcation_202401_lessClusters.h5ad")

del adata.obs['doublet_scores']
del adata.obs['predicted_doublets']
del adata.layers
del adata.uns
del adata.varm
del adata.obsp
adata
```

Out[5]:

```
AnnData object with n_obs × n_vars = 10396 × 29143
    obs: 'sample', 'batch', 'group', 'n_genes_by_counts', 'total_counts', 'total_counts_mito', 'pct_counts_mito', 'n_counts', 'size_factors', 'leiden'
    var: 'gene_ids', 'feature_types', 'mito', 'n_cells_by_counts', 'mean_counts', 'pct_dropout_by_counts', 'total_counts', 'n_counts', 'highly_variable', 'means', 'dispersions', 'dispersions_norm'
    obsm: 'X_pca', 'X_pca_harmony', 'X_umap'
```

In [6]:

```
scv.utils.clean_obs_names(adata)
scv.utils.clean_obs_names(ldata)
adata = scv.utils.merge(adata, ldata)
scv.pl.proportions(adata)
```

In [7]:

```
scv.pp.moments(adata, n_pcs=20, n_neighbors=20)
scv.tl.velocity(adata)
```

```
WARNING: Did not normalize X as it looks processed already. To enforce normalization, set `enforce=True`.
Normalized count data: spliced, unspliced.
computing neighbors
    finished (0:00:23) --> added 
    'distances' and 'connectivities', weighted adjacency matrices (adata.obsp)
computing moments based on connectivities
    finished (0:00:09) --> added 
    'Ms' and 'Mu', moments of un/spliced abundances (adata.layers)
computing velocities
    finished (0:00:31) --> added 
    'velocity', velocity vectors for each individual cell (adata.layers)
```

In [8]:

```
scv.tl.velocity_graph(adata)
```

```
computing velocity graph (using 1/56 cores)
```

```
  0%|          | 0/10396 [00:00<?, ?cells/s]
```

```
    finished (0:00:12) --> added 
    'velocity_graph', sparse matrix with cosine correlations (adata.uns)
```

In [9]:

```
scv.tl.recover_dynamics(adata)
scv.tl.velocity(adata, mode='dynamical')
scv.tl.velocity_graph(adata)
adata.write('./results/scvelo_dynamic_data.h5ad', compression='gzip')
```

```
recovering dynamics (using 1/56 cores)
```

```
  0%|          | 0/486 [00:00<?, ?gene/s]
```

```
    finished (0:08:43) --> added 
    'fit_pars', fitted parameters for splicing dynamics (adata.var)
computing velocities
    finished (0:00:36) --> added 
    'velocity', velocity vectors for each individual cell (adata.layers)
computing velocity graph (using 1/56 cores)
```

```
  0%|          | 0/10396 [00:00<?, ?cells/s]
```

```
    finished (0:00:10) --> added 
    'velocity_graph', sparse matrix with cosine correlations (adata.uns)
```

In [10]:

```
adata
```

Out[10]:

```
AnnData object with n_obs × n_vars = 10396 × 29143
    obs: 'sample', 'batch', 'group', 'n_genes_by_counts', 'total_counts', 'total_counts_mito', 'pct_counts_mito', 'n_counts', 'size_factors', 'leiden', 'sample_batch', 'initial_size_unspliced', 'initial_size_spliced', 'initial_size', 'velocity_self_transition'
    var: 'gene_ids', 'feature_types', 'mito', 'n_cells_by_counts', 'mean_counts', 'pct_dropout_by_counts', 'total_counts', 'n_counts', 'highly_variable', 'means', 'dispersions', 'dispersions_norm', 'Accession', 'Chromosome', 'End', 'Start', 'Strand', 'sample', 'velocity_gamma', 'velocity_qreg_ratio', 'velocity_r2', 'velocity_genes', 'fit_alpha', 'fit_beta', 'fit_gamma', 'fit_t_', 'fit_scaling', 'fit_std_u', 'fit_std_s', 'fit_likelihood', 'fit_u0', 'fit_s0', 'fit_pval_steady', 'fit_steady_u', 'fit_steady_s', 'fit_variance', 'fit_alignment_scaling', 'fit_r2'
    uns: 'neighbors', 'velocity_params', 'velocity_graph', 'velocity_graph_neg', 'recover_dynamics'
    obsm: 'X_pca', 'X_pca_harmony', 'X_umap'
    varm: 'loss'
    layers: 'matrix', 'ambiguous', 'spliced', 'unspliced', 'Ms', 'Mu', 'velocity', 'variance_velocity', 'fit_t', 'fit_tau', 'fit_tau_', 'velocity_u'
    obsp: 'distances', 'connectivities'
```

In [11]:

```
#adata = scv.read('/mnt/dzl_bioinf/gaedckes/scRNASeq/Macrophagen_AGLachmann_202311/velocity/scvelo_dynamic_data.h5ad')
scv.pl.velocity_embedding_stream(adata, basis='umap', color=["leiden","sample"])# save="velocity_dynamic")
```

```
computing velocity embedding
    finished (0:00:03) --> added
    'velocity_umap', embedded velocity vectors (adata.obsm)
```

In [12]:

```
scv.pl.velocity_embedding_stream(adata, basis='umap', color="leiden", 
                                 legend_loc= "right margin"),# save="velocity_dynamic2", 
                            #    xlim = [-0.5,15], ylim=[-7,9])
```

Out[12]:

```
(None,)
```

In [13]:

```
# latent time
scv.tl.latent_time(adata)
scv.pl.scatter(adata, color='latent_time', color_map='gnuplot', size=80)# save="velocity_dynamic_latenttime")
```

```
computing terminal states
    identified 3 regions of root cells and 1 region of end points .
    finished (0:00:00) --> added
    'root_cells', root cells of Markov diffusion process (adata.obs)
    'end_points', end points of Markov diffusion process (adata.obs)
computing latent time using root_cells as prior
    finished (0:00:13) --> added 
    'latent_time', shared time (adata.obs)
```

In [14]:

```
top_genes = adata.var['fit_likelihood'].sort_values(ascending=False).index[:300]
adata.var['fit_likelihood'].sort_values(ascending=False).to_csv("/mnt/dzl_bioinf/gaedckes/scRNASeq/Macrophagen_AGLachmann_202311/velocity/topgenes_latentime.csv")
```

In [15]:

```
scv.tl.rank_dynamical_genes(adata, n_genes=100, groupby="leiden")   
#df = scv.DataFrame(adata.uns['rank_dynamical_genes']['names'])
#df.to_csv("/mnt/dzl_bioinf/gaedckes/scRNASeq/Bcell/results/rankdynamicgenes_latentime.csv")
#df.head(20)
```

```
ranking genes by cluster-specific likelihoods
    finished (0:00:04) --> added 
    'rank_dynamical_genes', sorted scores by group ids (adata.uns)
```

In [16]:

```
scv.pl.heatmap(adata, var_names=top_genes, sortby='latent_time', col_color='leiden',
               n_convolve=100)#              save="velocity_dynamic_heatmap_latenttime")
```

In [17]:

```
top_genes = adata.var['fit_likelihood'].sort_values(ascending=False).index
scv.pl.scatter(adata, basis=top_genes[:15], ncols=5, frameon=False, color="leiden")
              #save="velocity_dynamic_scatter")
```

In [23]:

```
scv.pl.velocity(adata, top_genes[:5], color="leiden", ncols=1)#, save="velocity_gene")
```

In [24]:

```
scv.tl.paga(adata, groups="leiden")
scv.pl.paga(adata, frameon=False, add_outline =True)#, save="paga")
scv.pl.paga(adata, basis='umap', size=50, alpha=.2,min_edge_width=2, node_size_scale=1.5)#, save="paga2")
scv.pl.paga(adata, basis='umap', min_edge_width=1)#,  save="paga3")
scv.pl.paga(adata, basis='umap')#, save="paga4")
```

```
running PAGA using priors: ['velocity_pseudotime']
    finished (0:00:02) --> added
    'paga/connectivities', connectivities adjacency (adata.uns)
    'paga/connectivities_tree', connectivities subtree (adata.uns)
    'paga/transitions_confidence', velocity transitions (adata.uns)
```

```
/mnt/dzl_bioinf/exec/miniconda/miniconda3/envs/scRNA2023/lib/python3.9/site-packages/networkx/convert.py:158: DeprecationWarning: 

The scipy.sparse array containers will be used instead of matrices
in Networkx 3.0. Use `from_scipy_sparse_array` instead.
  return nx.from_scipy_sparse_matrix(data, create_using=create_using)
```

```
WARNING: Invalid color key. Using grey instead.
```

```
/mnt/dzl_bioinf/exec/miniconda/miniconda3/envs/scRNA2023/lib/python3.9/site-packages/networkx/convert.py:158: DeprecationWarning: 

The scipy.sparse array containers will be used instead of matrices
in Networkx 3.0. Use `from_scipy_sparse_array` instead.
  return nx.from_scipy_sparse_matrix(data, create_using=create_using)
```

```
WARNING: Invalid color key. Using grey instead.
```

```
/mnt/dzl_bioinf/exec/miniconda/miniconda3/envs/scRNA2023/lib/python3.9/site-packages/networkx/convert.py:158: DeprecationWarning: 

The scipy.sparse array containers will be used instead of matrices
in Networkx 3.0. Use `from_scipy_sparse_array` instead.
  return nx.from_scipy_sparse_matrix(data, create_using=create_using)
```

```
WARNING: Invalid color key. Using grey instead.
```

```
/mnt/dzl_bioinf/exec/miniconda/miniconda3/envs/scRNA2023/lib/python3.9/site-packages/networkx/convert.py:158: DeprecationWarning: 

The scipy.sparse array containers will be used instead of matrices
in Networkx 3.0. Use `from_scipy_sparse_array` instead.
  return nx.from_scipy_sparse_matrix(data, create_using=create_using)
```

```
WARNING: Invalid color key. Using grey instead.
```

```
/mnt/dzl_bioinf/exec/miniconda/miniconda3/envs/scRNA2023/lib/python3.9/site-packages/networkx/convert.py:158: DeprecationWarning: 

The scipy.sparse array containers will be used instead of matrices
in Networkx 3.0. Use `from_scipy_sparse_array` instead.
  return nx.from_scipy_sparse_matrix(data, create_using=create_using)
```

# non dynamic mode¶

In [24]:

```
scv.tl.velocity(adata, mode='stochastic')
scv.pl.velocity_embedding_stream(adata, basis='umap', color=["leiden","sample"])# save="velocity_dynamic")
```

```
computing velocities
    finished (0:00:27) --> added 
    'velocity', velocity vectors for each individual cell (adata.layers)
computing velocity embedding
    finished (0:00:03) --> added
    'velocity_umap', embedded velocity vectors (adata.obsm)
```

In [25]:

```
scv.pl.velocity(adata, ["ZEB2"], color="leiden")#, save="velocity_gene")
```

In [26]:

```
scv.tl.paga(adata, groups="leiden")
scv.pl.paga(adata, frameon=False, add_outline =True)#, save="paga")
scv.pl.paga(adata, basis='umap', size=50, alpha=.2,min_edge_width=2, node_size_scale=1.5)#, save="paga2")
scv.pl.paga(adata, basis='umap', min_edge_width=1)#,  save="paga3")
scv.pl.paga(adata, basis='umap')#, save="paga4")
```

```
running PAGA using priors: ['velocity_pseudotime']
    finished (0:00:02) --> added
    'paga/connectivities', connectivities adjacency (adata.uns)
    'paga/connectivities_tree', connectivities subtree (adata.uns)
    'paga/transitions_confidence', velocity transitions (adata.uns)
```

```
/mnt/dzl_bioinf/exec/miniconda/miniconda3/envs/scRNA2023/lib/python3.9/site-packages/networkx/convert.py:158: DeprecationWarning: 

The scipy.sparse array containers will be used instead of matrices
in Networkx 3.0. Use `from_scipy_sparse_array` instead.
  return nx.from_scipy_sparse_matrix(data, create_using=create_using)
```

```
WARNING: Invalid color key. Using grey instead.
```

```
/mnt/dzl_bioinf/exec/miniconda/miniconda3/envs/scRNA2023/lib/python3.9/site-packages/networkx/convert.py:158: DeprecationWarning: 

The scipy.sparse array containers will be used instead of matrices
in Networkx 3.0. Use `from_scipy_sparse_array` instead.
  return nx.from_scipy_sparse_matrix(data, create_using=create_using)
```

```
WARNING: Invalid color key. Using grey instead.
```

```
/mnt/dzl_bioinf/exec/miniconda/miniconda3/envs/scRNA2023/lib/python3.9/site-packages/networkx/convert.py:158: DeprecationWarning: 

The scipy.sparse array containers will be used instead of matrices
in Networkx 3.0. Use `from_scipy_sparse_array` instead.
  return nx.from_scipy_sparse_matrix(data, create_using=create_using)
```

```
WARNING: Invalid color key. Using grey instead.
```

```
/mnt/dzl_bioinf/exec/miniconda/miniconda3/envs/scRNA2023/lib/python3.9/site-packages/networkx/convert.py:158: DeprecationWarning: 

The scipy.sparse array containers will be used instead of matrices
in Networkx 3.0. Use `from_scipy_sparse_array` instead.
  return nx.from_scipy_sparse_matrix(data, create_using=create_using)
```

```
WARNING: Invalid color key. Using grey instead.
```

```
/mnt/dzl_bioinf/exec/miniconda/miniconda3/envs/scRNA2023/lib/python3.9/site-packages/networkx/convert.py:158: DeprecationWarning: 

The scipy.sparse array containers will be used instead of matrices
in Networkx 3.0. Use `from_scipy_sparse_array` instead.
  return nx.from_scipy_sparse_matrix(data, create_using=create_using)
```

# Save notebook as html¶

In [26]:

```
%%bash
jupyter nbconvert --to html_toc Velocity_analysis.ipynb --ExtractOutputPreprocessor.enabled=False
mv Velocity_analysis.html ./results/Velocity_analysis_$(date +%Y%m%d).html
```

```
Numba: Attempted to fork from a non-main thread, the TBB library may be in an invalid state in the child process.
[NbConvertApp] WARNING | Config option `kernel_spec_manager_class` not recognized by `NbConvertApp`.
[NbConvertApp] Converting notebook Velocity_analysis.ipynb to html_toc
[NbConvertApp] Writing 11402558 bytes to Velocity_analysis.html
```
